# Identification of genes associated with the regulation of cold tolerance and the RNA movement in the grafted apple

**DOI:** 10.1038/s41598-023-38571-2

**Published:** 2023-07-18

**Authors:** Youngsuk Lee, Nam V. Hoang, Van Giap Do, Toshi M. Foster, Tony K. McGhie, Seonae Kim, Sang Jin Yang, Ju-Hyeon Park, Jongsung Park, Ji-Young Lee

**Affiliations:** 1 School of Biological Sciences, College of National Science, Seoul National University, 1 Gwanak-Ro, Gwanak-Gu, Seoul, 08826 South Korea; 2 Apple Research Institute, National Institute of Horticultural and Herbal Science, Rural Development Administration, 107, Soboangye-Ro, Gunwi, 39000 South Korea; 3 Wageningen University & Research, Droevendaalsesteeg 1, 6708 PB, Wageningen, The Netherlands; 4 The New Zealand Institute for Plant and Food Research Limited, 55 Old Mill Road, Motueka, New Zealand; 5 The New Zealand Institute for Plant and Food Research Limited, Private Bag 11600, Palmerston North, New Zealand

**Keywords:** Molecular biology, Plant sciences

## Abstract

In grafted apple, rootstock-derived signals influence scion cold tolerance by initiating physiological changes to survive over the winter. To understand the underlying molecular interactions between scion and rootstock responsive to cold, we developed transcriptomics and metabolomics data in the stems of two scion/rootstock combinations, ‘Gala’/‘G202’ (cold resistant rootstock) and ‘Gala’/‘M9’ (cold susceptible rootstock). Outer layers of scion and rootstock stem, including vascular tissues, were collected from the field-grown grafted apple during the winter. The clustering of differentially expressed genes (DEGs) and gene ontology enrichment indicated distinct expression dynamics in the two graft combinations, which supports the dependency of scion cold tolerance on the rootstock genotypes. We identified 544 potentially mobile mRNAs of DEGs showing highly-correlated seasonal dynamics between scion and rootstock. The mobility of a subset of 544 mRNAs was validated by translocated genome-wide variants and the measurements of selected RNA mobility in tobacco and *Arabidopsis*. We detected orthologous genes of potentially mobile mRNAs in *Arabidopsis thaliana*, which belong to cold regulatory networks with RNA mobility. Together, our study provides a comprehensive insight into gene interactions and signal exchange between scion and rootstock responsive to cold. This will serve for future research to enhance cold tolerance of grafted tree crops.

## Introduction

The cold response is essential for plants to survive during the winter or an unusually chilly period. Physiological acclimation to cold includes the modification of membrane fluidity, changes in metabolite accumulation, and adjustment of activity of intracellular organelles such as chloroplasts^1^. The cold response in plants relies on C-repeat binding factors (CBFs) that promote the expression of downstream *COLD-RESPONSIVE* (*COR*) genes^2,3^. Abscisic acid (ABA) initiates an early cold response by activating CBFs. Jasmonic acid (JA) also enhances cold tolerance by inhibiting the repression of jasmonate zim-domain (JAZ) on CBF expression^4–6^.

Perennial tree species adapted to temperate climates have genetic programs that allow them to survive over the cold winter. In late autumn/early winter, the metabolic signals in vascular tissues generally flow from shoot to root as the tree enters the dormancy to overcome external non-freezing cold temperature, a process known as cold acclimation^7,8^. In the middle of winter, plants stop their growth and development, which is called endodormancy. After fulfilling the chill requirement, the transition to ecodormancy, a growth-competent state, occurs^9^. In late winter/early spring, trees resume growth by activating various signals moving from root to shoot through vascular tissues, depending on de-acclimation status to the cold, i.e., losing tolerance to low temperatures^10^. In apples, for example, the changes in the expression of dormancy-related genes such as *DORMANCY ASSOCIATED MADS-BOX (DAM)* or *DEHYDRIN (DHN)*^11,12^ occur when a plant enters into or exits from dormancy.

This cold response mechanism is crucial in many tree crops routinely grafted onto clonal rootstocks. Rootstocks enhance the beneficial traits of scion cultivars, such as growth vigor, disease resistance, or tolerance to abiotic stresses^13–16^.

Apple (*Malus* × *domestica* Borkh.) is a major tree crop that occupies a significant portion of the world's fruit industry. Over the winter, changes in cold temperatures can damage stem tissues near the graft junction, allowing pathogen infection that can cause abnormal tree growth or death. The trait of rootstock could influence the overall tolerance of apple scion to cold stress during the winter. One of the most widely used apple rootstocks is ‘Malling 9’ (‘M9’). M9 rootstock series are known to be susceptible to cold, whereas other rootstocks such as ‘Geneva’ and ‘Budagovsky’ series are cold tolerant^17–19^. However, little is known about molecular mechanisms underlying the long-distance mobile signals between rootstock and scion and the interactions between the two in response to cold.

We hypothesized that the differences in cold tolerance of rootstocks would affect that of scion and that this might be due to mobile signals derived from a rootstock. These mobile signals might directly enhance cold tolerance pathways or indirectly contribute through some intermediate signaling. To test this hypothesis, we investigated the molecular signals in the stem vasculature of scion and rootstock in field-grown grafted apple trees over the winter. We profiled transcriptomes in ‘Gala’ scions grafted and their two rootstocks, each of which is cold-resistant and susceptible. Transcriptome data allowed us to identify 544 potentially mobile mRNAs that might be responsible for the differential responses to cold stress in scions depending on rootstocks. We also found genes associated with cold acclimation and de-acclimation, expressed explicitly in either cold-tolerant or susceptible rootstocks. Our results provide a novel insight into cold signaling between rootstock and scion and serve as valuable resources for future research to enhance cold tolerance in apples.

## Materials and methods

### Plant materials and tissue collections for transcriptomics and metabolomics data

**Figure 1:**
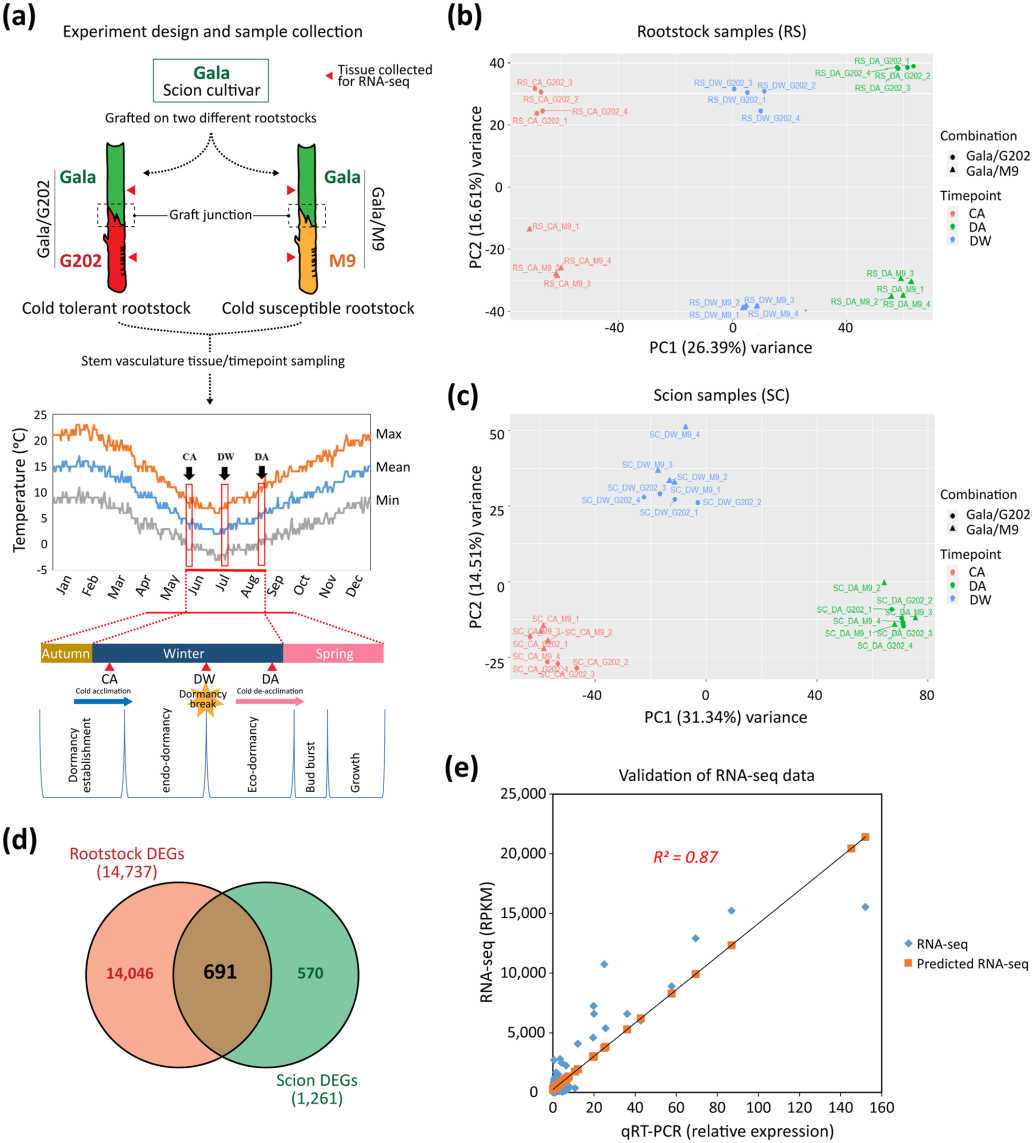
RNA-seq experiment design and DEG analysis. (**a**) A scheme for cold tolerance signaling study. Stem vasculature tissues of ‘Gala’ apple scion grafted onto two contrasting rootstock cultivars showing a different level of cold tolerance (‘G202’, ‘M9’) were sampled at three winter stages: CA (early winter/cold acclimation, 6th June), DW (deep winter, 17th July), DA (late winter/cold de-acclimation, 26th August). (**b**,**c**) Principal component analysis of RNA-seq expression data. Top 5000 genes listed according to the variance rank were chosen as informative genes and used for PCA analysis: (**b**) rootstock (RS) and **(c)** scion (SC). (**d**) Venn diagram of DEGs between scion and rootstock with a criterion of |fold change|≥ 1.5 FDR ≤ 0.05. (**e**) RNA-seq validation by qRT-PCR. A high significant correlation (*r* ≥ 0.9) was made between RNA-seq result (RPKM) and qPCR.

This study used two types of grafted trees: ‘Gala’ scion grafted to either cold-resistant ‘Geneva 202’ (‘G202’) rootstocks (‘Gala’/‘G202’) or cold-susceptible ‘M9 Pajam 2 (Cepiland)’ rootstocks (‘Gala’/‘M9’). These two dwarfing rootstocks are similar in their tree size and commercially available to produce grafted apple trees in New Zealand. One-year-old grafted trees (Waimea Nurseries, Appleton, NZ) were planted in August 2018 in a randomized block design at the Plant and Food Research Clyde research station (45.2°S, 169.3°E) in the South Island of New Zealand. Trees were grown under standard orchard conditions for ten months before tissue sampling began. The outer bark was removed with a razor, and then vascular-enriched tissues consisting of vascular cambium, phloem, sapwood xylem were collected 10 cm above and below the graft junction and immediately frozen in liquid nitrogen. Scion and rootstock samples were collected from six trees for each biological replicate at three different time points in the Year 2019 (Fig. 1a). These time points were early winter/cold acclimation (CA, 6th June), deep winter (DW, 17th July), and late winter/cold de-acclimation (DA, 26th August). The information for those sampling dates was matched to days before full bloom (DBFB) using the approximate day of full bloom for adult ‘Gala’ apple trees observed at the Clyde research station (Table S1). The time of dormancy release for ‘Gala’ was estimated based on the chilling unit accumulation models, models broadly applied to deciduous fruit crops^20,21^, combined with the public meteorological data at Clyde, provided by CustomWeather (https://www.timeanddate.com/weather) (Method S1, Fig. S1).

### Electrolyte leakage rate (ELR) measurement of apple genotypes

To assess the cold tolerance of each apple genotype used in this study, we first measured their ELR. 20 cm long stem segments collected from one-year-old branches of each genotype (‘Gala’, ‘G202’ and ‘M9’) were stored in closed 50 ml falcon tubes and placed onto a programmable thermo-controller DS-8504 M-S for cold treatment (Daewon Science, Seoul, Korea). For each genotype, six biological replicates were collected and analyzed. Samples were first treated with cold temperatures successively for 24 h at 4, − 4, and − 9 °C: six samples at 4 °C for 24 h; six samples at 4 °C for 24 h and then − 4 °C for 24 h; six samples at 4 °C for 24 h, − 4 °C for 24 h and then − 9 °C for 24 h. The temperature was lowered at a rate of 5 °C/h. We measured the electrical conductivity (EC) value to assess the levels of cold damage as previously studied^22^. Samples treated at each chilling/freezing temperature were cut into 5 mm pieces. Each 1.5 g weighted section was mixed with 15 ml distilled water and then placed onto a shaking incubator at 24 °C and 150r/min for 22 h. After the incubation, the initial EC was measured using an electric conductivity meter CON 11 (Eutech Instruments, Singapore). Samples were boiled in a water bath for 20 min to kill the tissues and cooled down for 2 h at room temperature. Then, the maximal EC was measured. Finally, the initial and maximum conductivity ratio was calculated to assess ELR at each chilling/freezing point.

### RNA, DNA sequencing, bioinformatics analyses, and single nucleotide polymorphism (SNP) detection

Total RNA was extracted from vascular-enriched tissues of ‘Gala’/‘G202’ and ‘Gala’/‘M9’ grafted apple trees using a modified CTAB method^23^. Based on RNA quality measured using Agilent 2100 bioanalyzer (Palo Alto, CA, USA), RNA samples with a RIN of 7 or above were sent to CnK genomics (South Korea) for sequencing. RNA-seq libraries were prepared using the Illumina TruSeq Stranded mRNA sample preparation kit (San Diego, CA, USA), and a total of 48 libraries were constructed using an Illumina Novaseq 6000 platform (San Diego, CA, USA) with 2 × 101-bp paired-end (PE) reads (Table S2). Additionally, we conducted whole-genome sequencing (WGS) of three genotypes (‘Gala’, ‘G202’, and ‘M9’) for SNP analysis to detect mobile mRNAs. Details of NGS analysis, data processing, and SNP detection are in Supporting information Method S2 and Table S3.

### Quantitative reverse transcription PCR (qRT-PCR)

To validate the RNA-seq results, qRT-PCR was carried out using LightCycler 480 SYBR Green I Master mix (Roche, Germany) on a Roche 480 LightCycler (Basel, Switzerland). Details of gene selection, primer design, and experiment conditions are provided in Supporting information Method S2 and Table S4.

### Functional study of MdTSJT1 mRNA mobility in response to cold using transiently overexpressing tobacco

To explore the mobility pattern of *MdTSJT1* mRNA under cold stress, we conducted an *Agrobacterium*-mediated infiltration assay using transient overexpression tobacco (*Nicotiana benthamiana*). For infiltration, the synthesized CDS of *MdTSJT1* (1170 bp) was inserted into the expression vector pCAMBIA1300 (CAMBIA, Canberra, ACT, Australia). The vector construct MdTSJT1::1300 was transformed into *A. tumefaciens* EHA105. To examine the mobility pattern in response to cold, the cold-dependent mRNA movement tendency in the stem toward the up-/downward direction was compared with the following calculation formula:

$${DM}_{(stem)}=\frac{{S}_{b}-{S}_{a}}{\left|{S}_{a}+{S}_{b}\right|} ,{ S}_{a/b}=\left[{RE}_{a/b}\left({T}_{n}\right)-{RE}_{a/b}({T}_{1})\right]/{RE}_{a/b}\left({T}_{1}\right),$$

where ${DM}_{(stem)}$ (degree of mRNA mobility in stem) represents the tendency of bidirectional movement associated with cold based on the relative abundance of normalized mRNA expression between two stem tissues above/below adjacent petiole of infiltrated leaf (e.g., if ${S}_{b}$ were more significant than ${S}_{a}$, the cold-dependent mRNA movement is considered presumably as upward and vice versa for the opposite case);${S}_{a/b}$ stands for the normalized transcript abundance in stem tissue above/below adjacent petiole; ${RE}_{a/b}$ as relative expression value of *MdTSJT1* against *NbFBOX* (Niben.v0.3.Ctg24993647), a tobacco housekeeping gene used as a reference gene in stem tissue above/below adjacent petiole for qRT-PCR calculation^24^; ${T}_{n}$ as each cold stage (${T}_{2}$ − ${T}_{4}$); ${T}_{1}$ as the time before cold treatment. Details about agroinfiltration into tobacco and short-term cold experiment are in Supporting information Method S3.

### The phenotypic assessment of cold tolerance in *Arabidopsis* genotypes and the functional assay of *Arabidopsis* grafting to explore AtVNI2 RNA movement responsive to cold

To explore the *AtVNI2* mRNA movement in response to cold, we conducted grafting experiments in *Arabidopsis thaliana* using the wild-type Col-0 and *vni2* loss-of-function mutant. A T-DNA insertion loss-of-mutant of *VNI2* (AT5G13180; ABRC stock number: salk_143793) seeds were obtained from ABRC (https://abrc.osu.edu/). Seeds for the line overexpressing *C-REPEAT BINDING FACTOR 3* (*CBF3)* and *cbf1 cbf2 cbf3* triple (*cbfs-1*) mutants were provided by Prof. Shuhua Yang (China Agricultural University, Beijing, China)^25^. The cold tolerance phenotypes were measured among four Arabidopsis genotypes (Col-0, *vni2*, *CBF3*, *cbfs-1*) including the survival rate, ELR and the compatible solute contents. Details of *Arabidopsis* seedlings and the phenotypic measurement are described in Supporting information Method S4. For the *Arabidopsis* grafting assay, twenty-day-old grafted *Arabidopsis* plants grown at 25 °C (14 days after grafting) were treated with cold stress. After decreasing to 4 °C, temperature was maintained for 7 d and then increased to 25 °C with a rate of 5 °C/h. Plants were grown under 16 h light/8 h dark photoperiod condition and sampled at designated time points: no cold treatment and the two cold stages: CA (24 h exposed to 4 °C), DA (24 h exposed to 25 °C after cold treatment). We collected samples from scion and rootstock tissues above/below the graft junction. Three biological replicates were collected at each time point. The relative expression levels of *AtVNI2* were analyzed by qRT-PCR and compared between scion and rootstock tissues of the grafted *Arabidopsis* across cold stages.

### Ultra-high-performance liquid chromatography-mass spectroscopy (UPLC-MS)

Each 0.05 g ground sample extracted from vascular-enriched tissues of ‘Gala’/‘G202’ and ‘Gala’/‘M9’ grafted apple trees from the same collection used for transcriptome analysis was extracted with 1 ml of ethanol: water: formic acid (80:20:1) solution and incubated for more than 12 h at 4 °C. There were six biological replicates, and the extracted samples were centrifuged at 15,000×*g* for 5 min. Supernatants were diluted tenfold with 100% methanol and analyzed in both positive and negative ionization modes. The metabolites were detected using UPLC-quadrupole-time-of-flight MS (UPLC-QTOF-MS) and labeled by accurate mass *m*/*z* and liquid chromatography retention time as previously described^26^.

### Compliance with international, national and/or institutional guidelines

The experimental research was carried out complying with relevant institutional, national, and international guidelines and legislation.

## Results and discussion

### Global transcriptome profile reveals seasonal and rootstock-genotypic differences between two apple graft combinations

In this study, we analyzed two types of grafted apple trees, ‘Gala’/’G202’ and ‘Gala’/’M9’. These consisted of the same scion genotype (‘Gala’) grafted to either cold-tolerant (‘G202’) or susceptible (‘M9’) rootstocks (Fig. 1a). The stem diameter did not show any significant difference (Student’s t-test, *p* > 0.05) between two graft combinations (Fig. S2), indicating that the stock genotype does not influence the scion growth. However, ELR measured at three chilling/freezing points (4, − 4, and − 9 °C) showed differences among three genotypes, ‘Gala’, ‘G202’, and ‘M9’ (Fig. S3). The scion genotype ‘Gala’ exhibited significantly high ELR values at all three points, suggesting that it is the least cold-tolerant among the three genotypes. ‘G202’ exhibited a considerably lower ELR at − 4 °C than ‘M9’. This result confirmed that the two rootstock genotypes, ‘G202’ and ‘M9’, contrast in cold tolerance.

To identify rootstock-derived signals that could influence cold tolerance of the scion, we compared gene expression and metabolite accumulation in the stem vasculature of scion and rootstock at three winter stages: CA (early), DW (middle), and DA (late) (see “Methods”). First, we obtained RNA-seq data from stem vasculature in the two apple graft combinations at three-time points: CA, DW, and DA. A total of 1298 million clean reads were obtained for 48 libraries from both rootstocks and scions (Tables S2, S5). Principal component analysis (PCA) revealed that the rootstock PCA explained the seasonal and genotypic differences, while scion PCA showed the seasonal difference (Fig. 1b,c, Note S1). This result is consistent with the fact that the rootstock samples are from two different genotypes, while the scion samples are from the same genotype. Then, we identified 14,737 rootstock DEGs and 1261 scion DEGs by comparing samples at each time point between two graft combinations with a threshold of |fold change|≥ 1.5 and FDR ≤ 0.05 (Fig. S4). Lists of DEGs at each time point are available in Table S6. As expected, rootstock DEGs (14,737) were much more than scion DEGs (1261), reflecting the difference in the two rootstock genotypes and their responses to cold stress. Nevertheless, we found a considerable number of scion DEGs (1261). These indicated that rootstocks strongly impact scion gene expression in response to cold stress. Furthermore, there were 691 DEGs shared between the rootstock DEGs and scion DEGs (Fig. 1d, Table S6). Finally, we performed qRT-PCR for 13 target genes which were randomly selected from the list of 691 shared DEGs between rootstocks and scions to validate the DEG analysis. The results showed a high correlation (*R*^*2*^ = *0.87, p* < *0.0001*) between data obtained from qRT-PCR and those of RPKM data from RNA-seq (Fig. 1e, Fig. S5, Table S7). These results suggest that our transcriptome data well represent the samples collected at different time points and from genotypes of contrasting cold tolerance.

### Differential expression of dormancy and cold-related markers denotes the seasonal response and the potential influence of rootstocks on scion cold tolerance

As the first step toward understanding the dynamics of our transcriptomes, we examined the expression of *SHORT VEGETATIVE PHASE* (*SVP*) and *DAM* genes^27^. These MADS-box genes are known to control the cycle of dormancy^11^.

The endodormancy and its breakage in our field-grown ‘Gala’ scion were expected to occur during the first two cold stages (CA and DW) (Fig. S1, see Method S1). Indeed, depending on the two graft combinations we investigated, DAMs and SVPs showed different expression dynamics. *MdDAMb* and *MdSVP* genes, functioning to maintain dormancy status, exhibited an upregulation at the DA stage in the ‘M9’ rootstock compared to ‘G202’ (Fig. S6a). In scion, *MdDAM* genes upregulated at CA gradually decreased their expression toward DA. This decrease in *MdDAM* expression was slow in scions of ‘Gala’/’M9’ compared to ‘Gala’/’G202’ (Fig. S6b). Furthermore, *MdSVPb* showed a dramatic increase in both rootstock and scion of 'Gala’/’M9’ at the DA stage. These results indicate that the duration of expression of genes regulating the dormancy/growth cycle is extended longer in cold-susceptible ‘Gala’/’M9’ relative to cold-tolerant ‘Gala’/’G202’, consistent with our expectations.

We also investigated the expression pattern of genes, which are known to be involved in the ICE-CBF cold tolerance pathway mediated by jasmonic acid signaling in apple^5^. In this analysis, some essential genes controlling cold tolerance signaling were upregulated in rootstock and downregulated in scion between two apple graft combinations. In early winter (CA), the expression of genes involved in cold tolerance (*MdCBF3, MdCBF4,* and *MdCOR47*) was downregulated in the ‘G202’ rootstock compared to ‘M9’, whereas their expression somewhat increased in the scion (Fig. S7). Consistent with this, the upstream negative regulators of the ICE-CBF pathway (*MdJAZ1, MdJAZ2*, and *MdMIEL1*) were downregulated in the ‘G202’ rootstock and upregulated in the ‘M9’ in late winter (DA). However, these expression patterns were the opposite in the scion. These results suggest that the cold response in rootstock may contribute to the signals in the scion for cold tolerance.

### Gene expression dynamics show rootstock-dependent responses to cold over the winter

**Figure 2:**
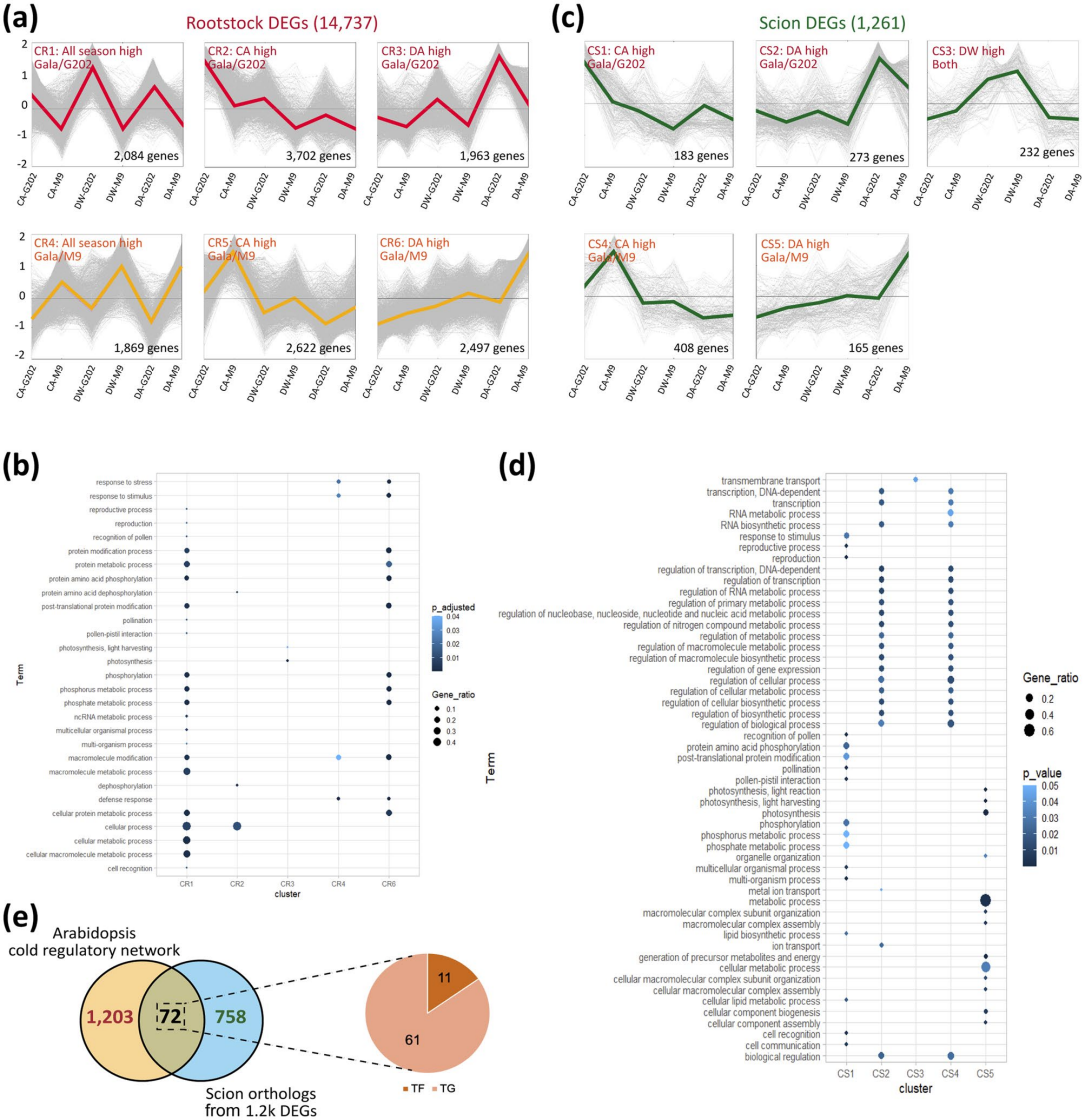

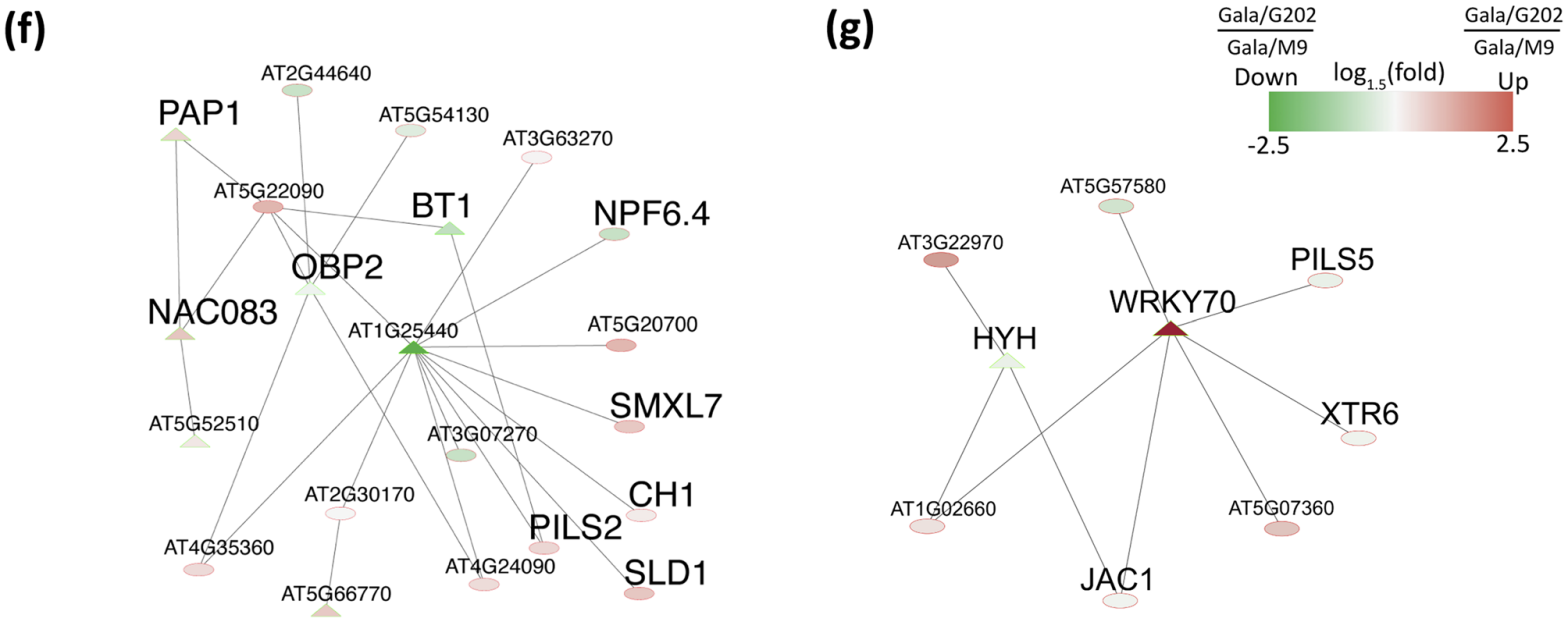
Expression dynamics of DEG clusters, functional annotation and comparative analysis of DEGs to *Arabidopsis* cold regulatory network. (**a**) K-means clustering of rootstock 14,747 DEGs (k = 6). (**b**) Gene ontology enrichment (biological process) of DEGs from five rootstock clusters. All clusters except CR5 (All high in ‘M9’) showed a significant GO terms with a criteria of FDR 0.05. (**c**) K-means clustering of scion 1261 DEGs (k = 5). (**d**) Gene ontology enrichment (biological process) of scion DEGs from with a criteria of *p* < 0.05. (**e**–**g**) Comparative analysis of scion DEGs to the cold regulatory network data conserved in *Arabidopsis*. (**e**) Venn diagram between scion orthologs and genes involved in cold regulatory network. 830 non-duplicated putative orthologs of *Arabidopsis* were obtained from 1251 scion DEGs and used for cross-species comparison. 72 scion DEGs were commonly found consisting of 11 transcription factors (TFs) and 61 targets (TGs). (**f**,**g**) Two sub-networks were constructed from the 72 scion DEGs found in cold regulatory network in *Arabidopsis*. The expression data at CA stage was used for visualization, as the reference network data was constructed from the expression profile of *Arabidopsis* exposed to non-freeze cold temperature, which conditions were similar to those of CA stage in our RNA-seq data. Expression ratios in scions of genes in these sub-networks (‘Gala’/‘G202’ over ‘Gala’/‘M9’ at CA undergoing cold acclimation) are color-coded. *CA* cold acclimation, *DW* deep winter, *DA* cold de-acclimation, *G202* ‘Gala’/‘G202’, *M9* ‘Gala’/‘M9’.

To understand the expression dynamics of rootstock genes over three winter time points, we conducted K-means clustering of the 14,737 rootstock DEGs. Among six gene clusters of rootstocks (CR1-CR6, details presented in Fig. S8a and Table S8), two clusters (CR1 and CR4) showed the expression dynamics unique to each rootstock genotype (‘G202’ and ‘M9’, respectively) across all the stages. In contrast, the other four reflected the dynamics specific to cold stages (CA and DA) and genotypes (Fig. 2a). Among gene clusters upregulated in cold-tolerant rootstock ‘G202’ (CR1-CR3), we found that the most enriched GO terms in CR1 (*FDR* < *0.05*) were related to modification of cellular protein and macromolecules (GO: 0006464, GO: 0043412) and cell recognition (GO: 0008037) (Fig. 2b). In clusters CR2 and CR3 (representing the seasonal dynamics in ‘G202’ rootstock), the protein dephosphorylation (GO: 0006470) was significantly enriched at first in early winter (CA), while photosynthesis (GO: 0015979) was enriched in late winter stage (DA). On the other hand, the ‘M9’ rootstock-specific cluster (CR4) not only revealed the enrichment of the modification of macromolecule (GO: 0043412) but also the response to stress (GO: 0006950). No significant enriched term was detected in ‘M9’ at the CA stage (CR5). However, in late winter DA, a wide range of enrichment for modification of cellular protein and macromolecules (GO: 0006464, GO: 0043412) and response to stress (GO: 0006950) were found in CR6, implying that cold-susceptible rootstock may tend to be more responsive to stress over the winter. Detailed GO enrichment analysis results are presented in Table S9. These data revealed that the functions of genes dynamically expressed over the winter are distinctive in two rootstock genotypes, which may subsequently influence the cold tolerance of scions.

In scion, the expression dynamics of 1261 DEGs, depending on the cold stages and rootstock genotypes, were categorized into five clusters (CS1-CS5; Figs. 2c, S8b, and Table S10). CS1 and CS2 consisted of genes upregulated in ‘Gala’/‘G202’ at CA and DA stages, respectively. Genes in CS4 and CS5 were upregulated in ‘Gala’/‘M9’ at CA and DA stages, respectively. CS3 contained genes highly expressed in DW in both graft combinations. Due to the small size of scion DEGs in each cluster compared to those of rootstock, we investigated the enriched GO terms in scion tissues using the uncorrected *p*-value cutoff of 0.05 (Fig. 2d, Table S11). In CS1 (up in ‘Gala’/‘G202’ at CA), the enrichment of lipid biosynthesis and metabolism (GO: 0008610, GO: 0044255), response to stimulus (GO: 0050896), and protein phosphorylation (GO: 0006468) were observed. CS2 (up in ‘Gala’/‘G202’ at DA), on the other hand, exhibited the enrichment of transcription regulation (GO: 0006335), RNA biosynthesis, and metabolism (GO: 0032774, GO: 0051252), but interestingly these terms were also found in CS4 (up in ‘Gala’/‘M9’ scion at CA). CS5 (up in ‘Gala’/‘M9’ at DA) showed the enriched GO terms for light harvesting of photosynthesis (GO: 0009765) and cellular macromolecular complex assembly (GO: 0034622). Since the same scion genotype ‘Gala’ was used for both graft combinations, the different gene expression dynamics and functional enrichment in the two scion samples collected from ‘Gala’/‘G202’ and ‘Gala’/‘M9’ are likely the results of rootstocks' influence on scions. The cold tolerance rootstock ‘G202’ may have contributed to the enrichment of GO terms related to response to the stimulus at CA and transcription regulation at DA in the ‘Gala’ scion. In contrast, cold-susceptible rootstock ‘M9’ contributed to the enrichment of photosynthesis-related genes and a slow response to cold in the ‘Gala’ scion.

### Comparative analysis with cold regulatory co-expression network in *Arabidopsis* implies candidate metabolism associated with scion cold tolerance that is potentially affected by rootstocks

In *Arabidopsis*, a co-expression network consisting of 1275 genes involved in the cold regulatory network has been reported^28^. This network, inferred from the gene expression changes in 10 Arabidopsis ecotypes exposed to non-freezing temperatures, likely reflects a cold acclimation process. To find the potential influence of rootstocks on scion cold tolerance, we compared the scion DEG with the putative orthologs of *Arabidopsis* in the cold regulatory networks. Among 1256 scion DEGs, 72 genes, including 11 transcription factors (TFs) and 61 targets (TGs), were found to be part of the cold regulatory interaction networks in *Arabidopsis* (Fig. 2e). Among these conserved 72 orthologs, 21 and 9 genes formed two sub-networks (Fig. 2f,g and Table S12).

We found that genes in two sub-networks might be related to the metabolism associated with scion cold tolerance during the early stage of cold acclimation. Genes were involved in growth regulation, nitrogen (N) depletion, and osmotic tolerance (Fig. 2f,g). In the first network, *NAC DOMAIN CONTAINING PROTEIN 83/VNI2* (hereafter *VNI2*), a TF that activates the expression of cold-regulated genes and represses vascular formation to protect against cellular damage under cold stress^29^, was mainly upregulated in the scion of ‘Gala’/‘G202’ having three edges. The expression of *SPHINGOID LCB DESATURASE 1* (*SLD1*) ortholog, encoding sphingolipid long-chain base (LCB) desaturase, was also upregulated. In *Arabidopsis*, *sld1* mutant showed cold-induced tissue damage with the chlorotic phenotype^30^, as LCB unsaturation contributes to cold tolerance^31,32^. Another notable player mediating growth regulation was *OCS BINDING FACTOR/OBF BINDING PROTEIN 2* (*OBP2*), one of hub TFs in the network, which promotes the radial growth of phloem^33^.

Next, orthologs involved in the regulation of nitrogen use efficiency (NUE) were found downregulated in ‘Gala’/‘G202’, including *BTB AND TAZ DOMAIN PROTEIN 1* (*BT1*), *NRT1/PTR FAMILY 6.4* (*NPF6.4*). Previous studies reported an increased NUE^34^ in the *AtBT1* mutant and an increase in the expression of *AtNPF6.4* in shoots under low nitrogen (N) conditions^35^. In that context, the downregulation of these two genes in the scion of ‘Gala’/‘G202’ compared to ‘Gala’/‘M9’ points to a relatively high tolerance of ‘Gala’/‘G202’ to N depletion possibly induced by the cold stress that leads to reduced uptake of nutrients^36–38^. On the other hand, *SLAC1 HOMOLOGUE 3* (*SLAH3*), which activates nitrate efflux anion channels and mediates stomatal closure in response to drought stress^39,40^, was upregulated. In addition to N depletion, response to osmotic stress can also be cold-inducible, causing cellular dehydration^36^. Among scion orthologs, *WRKY DNA-BINDING PROTEIN 70* (*WRKY70*) and *PECTIN METHYL-ESTERASE INHIBITOR 13* (*PMEI13*), both of which regulate osmotic tolerance, were upregulated in the scion of cold tolerant ‘Gala’/‘G202’^41,42^. Our comparative analysis of scion DEGs to a cold regulatory network in *Arabidopsis* further provides evidence for scion cold tolerance under the influence of rootstocks.

### Analysis of shared DEGs between rootstock and scion uncovers potentially mobile mRNAs through grafted stem vasculature along with the seasonal flow and cold tolerance

**Figure 3:**
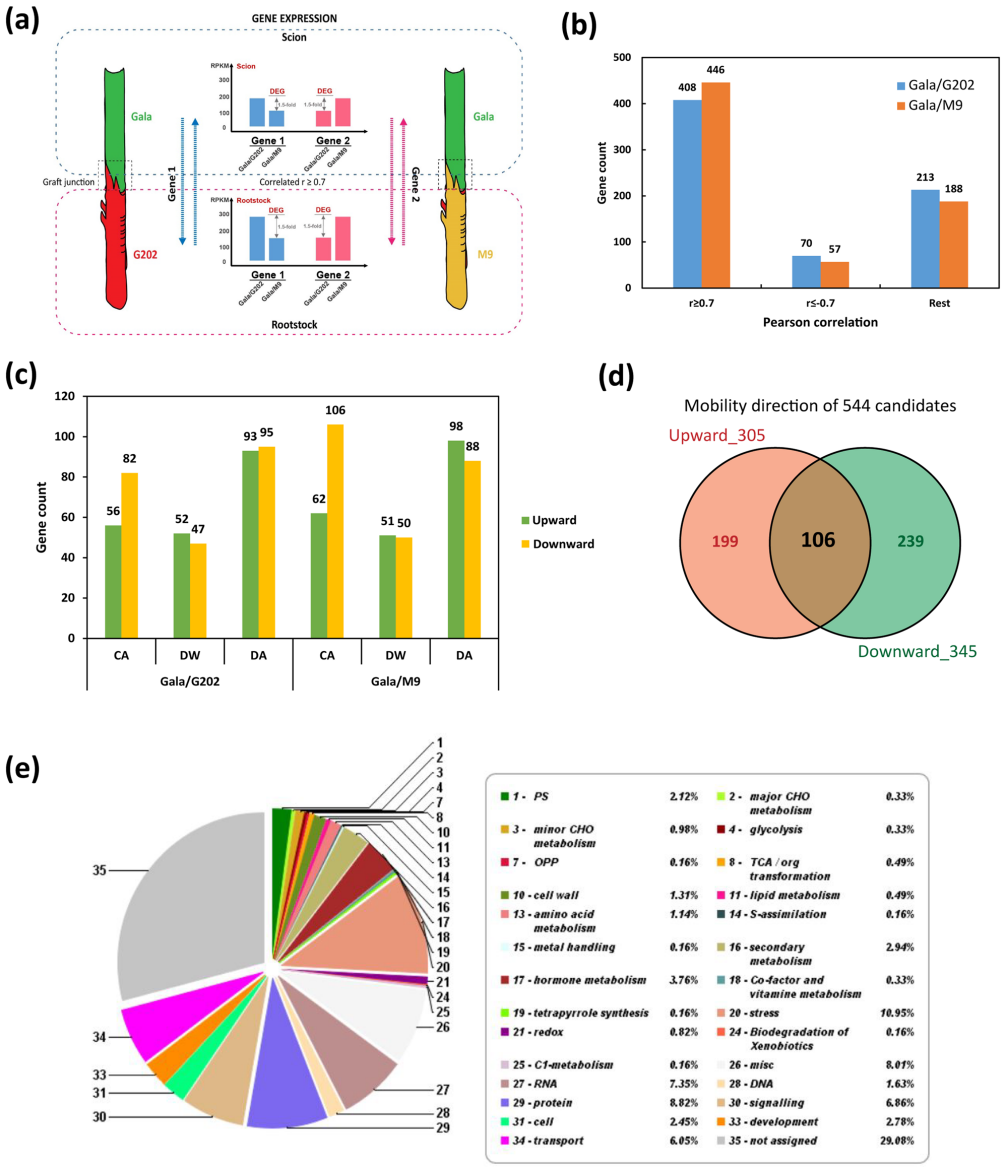
Investigation of highly-correlated genes for seasonal flow. (**a**) A scheme for the detection of mobile mRNAs of DEGs with seasonal flow. (**b**,**c**) Pearson correlation analysis on 691 DEG set. (**b**) Highly-correlated DEGs (*r* ≥ 0.7) between rootstock and scion were selected for further investigation. (**c**) Seasonal flow DEGs. Highly-correlated DEGs were divided into six groups by two flow directions: upwardly mobile (rootstock to scion) or downwardly mobile (scion to rootstock) combined with the cold stages. (**d**) Venn diagram of up- and downwardly mobile mRNAs of DEGs. (**e**) Functional categorization of highly-correlated 544 DEG set based on their DNA sequence homology using Mercator v3.6 tool. *CA* cold acclimation, *DW* deep winter, *DA* cold de-acclimation.

Distinct enrichment of genes in the same scion genotype grafted onto two different rootstocks indicated the presence of seasonal exchanges and interactions of signals between scions and rootstocks. As one form of exchanging signals, we explored the mobile mRNAs through the grafted stem vasculature in response to cold. If mRNAs were mobile, they would be present in both rootstock and scion and exhibit high correlations of RNA expression patterns in scions and in rootstocks (Fig. 3a). We used this criterion in our search for potentially mobile mRNAs, focusing on the 691 DEGs shared between DEGs from rootstock and scion data across all time points (Fig. 1d).

Through a Pearson’s correlation analysis of gene expression data between rootstocks and scions for each graft combination, we found that, out of 691 common DEGs, 552 genes showed a high correlation in their expression patterns (*r* ≥ 0.7). Among these, 302 genes were highly correlated in both ‘Gala’/‘G202’ and ‘Gala’/‘M9’, while 106 and 144 genes were unique to the respective combinations (Fig. 3b, Table S13). The correlated expression patterns of these genes could result from mRNA exchange between rootstock and scion through the graft junction in response to seasonal conditions.

Metabolic signals in plants move through the vasculature in response to the external changes in temperature during winter. These metabolic signals generally flow from shoot to root at the cold acclimation stage to store the assimilated nutrients and other signaling molecules in the root^43–45^. On the other hand, the movement of metabolites at the cold de-acclimation stage is from rootstock to scion to utilize them for restarting shoot growth^46,47^. RNA can be transported over long distances like other signaling molecules through the phloem^48^, and the movement could be concentration-dependent^49^. We divided the highly-correlated DEGs (*r* ≥ 0.7) into two directional groups of seasonal flow, upward (rootstock-to-scion) and downward (scion-to-rootstock), by comparing the relative RPKM ratio between rootstock and scion. For a given mRNA, if its expression in rootstock were higher than that in the scion, then its movement was considered upward. If the other way were true, then mRNA was treated as one moving downward. In this manner, we identified 138 (56/82), 99 (52/47), and 188 (93/95) mobile mRNAs (up-/downward) in CA, DW, and DA of ‘Gala’/‘G202’; and 168 (62/106), 101 (51/50), and 186 (98/88) mobile mRNAs (up-/downward) in CA, DW, and DA of ‘Gala’/‘M9’, respectively (Figs. 3c, Fig. S9a–c, Table S14). The non-redundant genes of mobile mRNAs summed from all stages and genotypes were 544 genes (Fig. 3d) and referred to as highly-correlated potentially mobile mRNAs. In general, there were more genes found in either CA or DA than in DW, reflecting the higher responsiveness of the plants in early or later winter compared to deep winter. In our analysis, genes showing directional flows of their mRNAs belonged to the functional categories including photosynthesis (bin 1), secondary metabolism (bin 16), hormone metabolism (bin 17), abiotic stress (bin 20), RNA (transcription factor) (bin 27), development (bin 33), signaling (bin 30), lipid metabolism (bin 11), and transport (bin 34) (Fig. 3e, Table S15). A part of a set of 544 genes encoding highly-correlated potential mobile mRNAs may represent signals in scion derived from rootstocks in response to cold. Notably, several GO terms including “*Photosynthesis*”, “*Phosphorylation*”, “*Shoot system morphogenesis*”, “*Xylem and phloem pattern formation*”, “*Regulation of hormone levels*” and “*Regulation of response to stimulus*”; and “*photosynthesis*” KEGG pathways were found to be enriched (FDR ≤ 0.05, Table S15).

### SNP analysis validates the rootstock-derived mobile mRNAs transported to scion in response to cold

**Figure 4:**
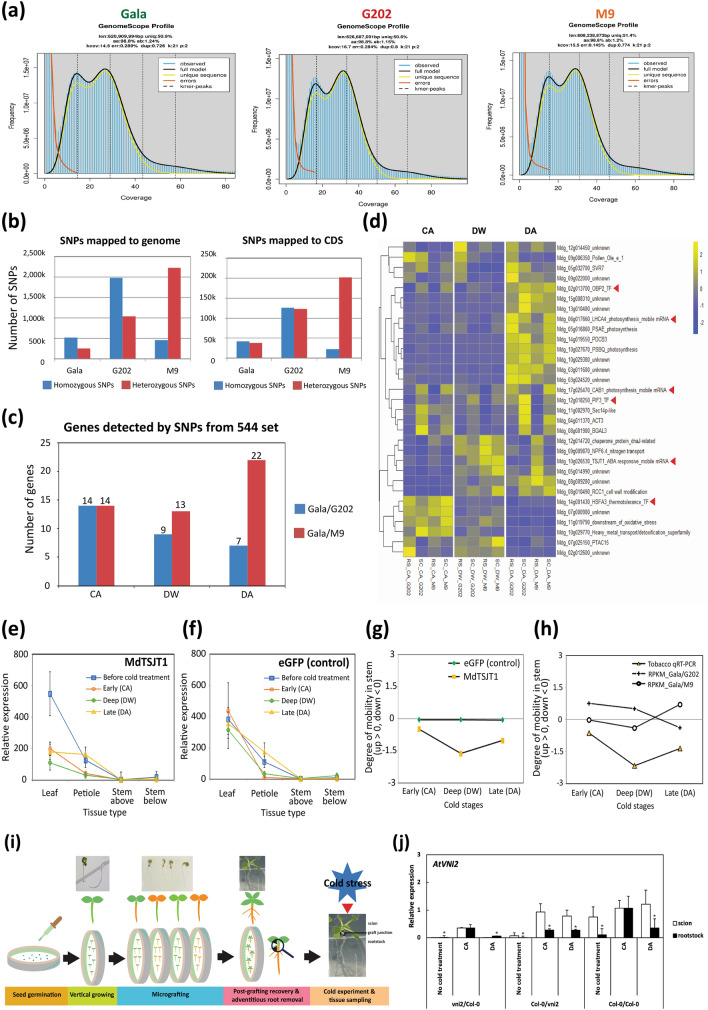
SNP detection and functional assays to validate the mRNA mobility associated with cold. (**a**–**d**) SNP detection to screen the mobility of rootstock-derived mRNAs to ‘Gala’ scion associated with the seasonal cold tolerance. (**a**) Estimation of heterozygosity of ‘Gala’, ‘G202’, ‘M9’ genotypes based on k-mer distribution. Heterozygous and homozygous peaks are shown from resequencing reads from paired-end libraries. (**b**) Genome-wide SNP calling from ‘Gala’, ‘G202’, ‘M9’ genotypes using resequencing data. (**c**) SNP detection of 544 highly-correlated seasonal flow DEGs. SNPs unique to each genotype were detected with the parameters of coverage 10, count 10, and frequency 20% in each cold stage. (**d**) Expression heatmap of 31 mobile mRNAs that contain rootstock-derived SNPs pooled from all winter stages. (**e**–**h**) Tobacco agroinfiltration assay. (**e**,**f**) Expression profile of genes in four tissue types of transiently overexpressing tobacco including the infiltrated leaf, petiole and the adjacent stem above/below the infiltrated leaf. Tissues were sampled at three cold stages (early, deep, late) following three days after agroinfiltration. (**e**) *MdTSJT1*; (**f**) *eGFP* (control). There were six to eight biological replicates. *NbFBOX* (Niben.v0.3.Ctg24993647) was used as reference gene for qRT-PCR calculation. (**g**) Degree of cold-dependent mRNA mobility in tobacco stem. (**h**) Comparison of degree of cold-dependent mobility of *MdTSJT1* between qRT-PCR of transiently overexpressing tobacco and RPKMs of RNA-seq data (upward > 0, downward < 0). (**i**,**j**) *Arabidopsis* grafting assay and the investigation of cold-dependent *AtVNI2* mRNA movement. (**i**) A scheme for *Arabidopsis* grafting for cold treatment. Six-day-old seedlings of wild-type or mutant medium-grown *Arabidopsis* plants were grafted. After post-grafting recovery, twenty-day-old grafted seedlings (14 days after grafting) were cold-treated and the whole scion/rootstock tissues above/below graft junction were collected to explore mRNA movement assay. (**j**) The relative expression of *AtVNI2.* Lines on the bars show standard error of the mean. *AtACTIN8* was used as reference gene for qRT-PCR calculation. Asterisk indicates significant difference at *p* < 0.05 by Student’s *t*-test. As expected, there was no expression data of *AtVNI2* detected in the *vni2*/*vni2* homografted *Arabidopsis*. *CA* cold acclimation, *DW* deep winter, *DA* cold de-acclimation, *RS* rootstock, *SC* scion, *G202* ‘Gala’/‘G202’, *M9* ‘Gala’/‘M9’.

We analyzed the rootstock genotype-specific SNPs (but not detected in scion genotype) obtained from WGS analysis of our transcriptome data. The WGS analysis suggested that the three apple genotypes used in this study exhibited a high level of heterozygosity (1.15–1.24%) (Fig. 4a,b, Table S16, Note S2). Utilizing SNPs, we aimed to seek evidence for the upward mobility of rootstock-derived mRNAs potentially affecting cold tolerance in the scion. We searched for the SNPs within the 544 mRNAs that were potentially mobile between rootstock and scion (Fig. 4c, Fig. S10). In this analysis, we discovered 31 mRNAs with rootstock genotype-specific SNPs, also present in the scion and among these 14 showed to have the mobility score of 3, indicating the high consistency of cold responsive RNA movement pattern concurrent with the result of seasonal flow DEG analysis (Table S17, Method S2). The low number of validated genes could be due to the high level of heterozygosity both in rootstock and scion genotypes since the analysis was limited to only a subset of homozygous SNPs found in the genome of rootstock genotypes but not in that of scion genotype. This SNP-based genome data analysis has been shown to help detect mobile RNAs in inter- or intra-species grafting^50^. Our experiments employed intra-species grafting, which limited the number of genes that could be validated using their SNPs since genes without any variants would be impossible to detect their mobility.

Nevertheless, functional annotation of potentially mobile mRNAs pointed to highly relevant genes among the 31 validated genes. These included transcription factors*, OBP2*, *PHYTOCHROME-INTERACTING FACTOR 3* (*PIF3*) and *HEAT SHOCK FACTOR A3* (*HSFA3*), as well as mobile mRNAs encoding LIGHT-HARVESTING CHLOROPHYLL-PROTEIN COMPLEX I SUBUNIT A4 (LHCA4), CHLOROPHYLL A/B BINDING PROTEIN 1 (CAB1), and stem-specific protein TSJT1 (Fig. 4d, Table S18). Some of potentially mobile vascular mRNAs might serve as the cold tolerance signals in scion.

### Validation of the cold-dependent mRNA movement in tobacco and *Arabidopsis*

To further infer whether potentially mobile mRNAs were actually mobile in response to cold, we selected a couple of genes to validate the cold-dependent RNA movement in tobacco and *Arabidopsis*.

First, we conducted agroinfiltration-based mobility assay in evolutionarily divergent *Nicotiana benthamiana*. We selected *MdTSJT1* for agroinfiltration since its mRNA seemed to be actively mobile for a following reason. Rootstock genotype-specific SNPs in *MdTSJT1* mRNA was observed in the scion RNA-seq reads in the CA, indicating that *MdTSJT1* mRNA is moving from the rootstock to the scion. This movement direction is against the general source-to-sink flow in the CA (early winter), which is from the scion to the rootstock (Table S19, Note S3). After short-term cold treatment followed by agroinfiltration, the mRNA gradient of *MdTSJT1* was formed across four tissues, successively from leaf to stem in transiently overexpressing tobacco. We selected *eGFP* agroinfiltrated tobacco lines as cold-independent control to discern the cold-dependent mobility patterns of *MdTSJT1* from a normal diffusion that occurs along the mRNA expression gradient. The mRNA level of both *MdTSJT1* and *eGFP* was highest in the infiltrated leaf. Subsequently, it decreased in adjacent petiole and the lowest in stem tissues across all cold stages, indicating the movement of mRNA according to its relative abundance among tissues (Fig. 4e,f). Previous studies demonstrated the majority of mRNA movement is predominantly driven by relative abundance. At the same time, there are still other possible mechanisms that could also trigger the transport of mRNAs across tissues against low-abundance conditions (i.e., a transcript that is functionally important and involved in signaling that has a specific transport system)^51,52^. In this study, we assumed relative abundance as the main driving force that induces mRNA movement.

The degree of mRNA mobility in stem, ${DM}_{ (stem)}$ was devised to assess the potential tendency of bidirectional cold-dependent movement of mRNA transported from infiltrated leaf into stem (see “Methods”). ${DM}_{(stem)}$ values of *eGFP* stayed close to zero throughout cold stages (− 0.06/− 0.06/− 0.07 at CA/DW/DA), reflecting little preference for up/down directionality and no relation to the cold-responsive movement of mRNA in the stem. By contrast, *MdTSJT1* showed a distinctive pattern of ${DM}_{(stem)}$ values over cold periods, showing a downward movement at CA (− 0.64), DW (− 2.16) and DA (− 1.35) (Fig. 4g). We then extended our analysis of ${DM}_{(stem)}$ to RNA-seq data by applying RPKM values of *MdTSJT1* in each apple graft combination. Interestingly, the pattern of ${DM}_{(stem)}$ of MdTSJT1 in tobacco was similar to that of cold susceptible ‘Gala’/‘M9’ than ‘Gala’/‘G202’ (Fig. 4h).

We also performed *Arabidopsis* grafting assay to demonstrate the cold-dependent RNA movement using the loss-of-function mutant of *VNI2* in *Arabidopsis thaliana* (*AtVNI2* hereafter) (Fig. 4i). We chose this mutant because *Mdg_13g010770* gene in apple homologous to *AtVNI2* exhibited the mRNA movement at CA and DA stages (Tables S14). Additionally, *AtVNI2* was found in the *Arabidopsis* cold regulatory network (Table S12). Consistent with this, the *vni2* mutant exhibited cold susceptible phenotypes: Its ELR was highest among the four genotypes used in our experiment, Col-0, *vni2*, *CBF3* (cold tolerant)^53^, and *cbfs-1* (cold susceptible)^25^, while its compatible solute and survival rate were lowest (Fig. S11, Note S4).

Then, using the *vni2* mutant, we produced scion/rootstock *Arabidopsis* grafts with the wild-type Col-0 and compared the relative expression level of *AtVNI2* after cold stress among different graft combinations by qRT-PCR. As expected, there was no expression of *AtVNI2* detected in the control *vni2*/*vni2* homograft. Interestingly, we found that *AtVNI2* expression was strongly induced after cold stress in both wild-type scion and *vni2* rootstock in the Col-0/*vni2* heterograft (Fig. 4j). In the wild-type Col-0/Col-0 homograft, the scion displayed the higher expression of *AtVNI2* than the rootstock without cold treatment; however, the *AtVNI2* expression was increased in rootstock to a similar level to that in scion after 24 h exposure to the cold (CA). Likewise, in the heterografted scion of *vni2*/Col-0, we observed that the *AtVNI2* expression was also increased to a similar level to that in rootstock at CA, implying its mRNA was synthesized from the wild-type rootstock and moved to *vni2* scion through the graft junction in response to cold. Moreover, in the rootstock of Col-0/*vni2*, the *AtVNI2* expression was induced in the two cold stages (CA and DA) but the expression was significantly lower than that in scion, which also supports its mRNA movement from the wild-type scion to the *vni2* rootstock recipient. Taken together, these results not only confirmed the potential mobility of mRNA proposed from apple transcriptome, but also imply the possibility that the mRNA movement is functionally associated with differential cold tolerance.

### Functional identification and comparative analysis of seasonal flow integrate mRNA exchange and interactive metabolic responses between rootstock and scion associated with cold tolerance

So far, our analyses only suggest the mobility of the 544 mobile mRNAs. To further scrutinize the relationship between cold tolerance and mRNA mobility, we compared the functional annotation of the 544 mobile mRNAs to the ortholog dataset of *Arabidopsis* that is known for mRNA mobility^52^. Among a total of 405 non-redundant *Arabidopsis* orthologs that correspond to the 544 potentially mobile mRNAs in apple, we found 48 genes (11.8%) were shared with 2006 genes expressing mobile mRNAs in Arabidopsis (Table S20). Then, the direction of the mobility of the 48 shared mobile mRNAs obtained from our seasonal flow analysis was compared to those in *Arabidopsis* (as the reference direction). Among 48 shared mobile mRNAs, 39 (75%) showed consistency in their mobility direction (the confidence level for directional consistency greater than or equal to 1), reinforcing the mobility of seasonal flow mRNAs regarding cold tolerance.

Notably, 39 genes confirmed with directional consistency of mRNAs were part of metabolic responses associated with cold tolerance, including photosynthesis/chlorophyll, growth regulation, osmotic/thermo-tolerance, nutrient transport, and secondary metabolism. Genes in photosynthesis/chlorophyll included *PHOTOSYSTEM I SUBUNIT H-2* (*PSAH2*) at DW and *CAB1*, *PHOTOSYSTEM I LIGHT HARVESTING COMPLEX GENE 3* (*LHCA3*), *LIGHT-HARVESTING-LIKE 3:1* (*LIL3:1*) at DA stage. Interestingly, the mobility of these mRNAs was all identified as downwardly mobile only in ‘Gala’/‘M9’. Given that chloroplast modulates cold response through reactive oxygen species (ROS) signaling and photosynthetic damage is also cold-inducible^54–56^, distinctive patterns of vascular mRNA movement involved in cold-responsive metabolism are likely due to different rootstocks. Similar to this, KEGG pathway analysis also suggested the significant enrichment of photosynthesis-related term and their relevant mRNAs orthologous to *Arabidopsis* among seasonal flow DEGs (Table S21).

Next, in the category of growth regulation, a couple of orthologs that play a role in meristem development and differentiation were found, including *JACKDAW* (*JKD*) and *BARELY ANY MERISTEM* 3 (*BAM3*) at DW. mRNA of *JKD,* encoding a TF of the zinc finger family that modulates stem cell differentiation^57^, was upwardly mobile in ‘Gala’/‘G202’ but downwardly mobile in ‘Gala’/‘M9’. On the other hand, *BAM3*, a CLAVATA-like leucine-rich repeat receptor-like kinase family required for phloem development^58^, was downwardly mobile in ‘Gala’/‘G202’, implying contrasting regulation of scion vascular development or growth between two graft combinations to withstand the cold. In addition, we identified several players involved in auxin signaling showing distinctive patterns of mRNA movements (mostly downward in ‘Gala’/‘G202’ and upward in ‘Gala’/‘M9’). This suggests that auxin metabolism was less active when scion was grafted onto cold-susceptible rootstock (Fig. S12, Note S5). Likewise, *SUPPRESSOR OF G2 ALLELE SKP 1* (*SGT1*), which mediates auxin response and temperature-responsiveness by interacting with heat shock protein^59^, was found as downwardly mobile in cold tolerant ‘Gala’/‘G202’ at DA.

As mentioned earlier, we found 72 scion DEGs involved in the cold regulatory network (Fig. 2f,g). Among them, 39 orthologs were involved in the seasonal flow of mRNA movement (Table S22). For the function of osmotic tolerance, in early winter (CA) *PMEI13* mRNA was upwardly mobile in cold tolerant ‘Gala’/‘G202’. On the other hand, in deep winter (DW), mRNA of *CYCLIC NUCLEOTIDE-GATED CATION CHANNEL 4 CNGC4* (*CNGC4*) also known as *DND2*, which regulates cation uptake in response to osmotic condition and disease resistance against *Pseudomonas syringae*^60–62^, showed a contrasting direction of mRNA movement (downwardly in ‘Gala’/‘G202’ and upwardly in ‘Gala’/‘M9’). Among cold-inducible defense responses^63^, the resistance to *Pseudomonas syringae* is critical for fruit tree crops to prevent bacterial canker symptoms that may cause stem tissue split, leading trees to death^64,65^. Considering these, the opposite flow of *MdCNGC4* mRNA may indicate the differential scion defense responses induced by cold. For thermotolerance, *PECTIN METHYLESTERASE 34* (*PME34*), a member of pectin methylesterase family known to modulate cell wall flexibility contributing to thermotolerance^66^, was found downwardly mobile in cold susceptible ‘Gala’/‘M9’ in both CA and DA stages, revealing a high thermosensitivity compared to cold tolerant ‘Gala’/‘G202’.

For secondary metabolism, *PAP1*, *CHIL*, and *DIHYDROFLAVONOL 4-REDUCTASE* (*DFR*) mediate flavonoid biosynthesis and accumulation. While *MdPAP1* was downwardly mobile in ‘Gala’/‘G202’ at CA, *MdDFR* and *MdCHIL* were upwardly mobile in ‘Gala’/‘M9’ at DW and DA, respectively. About nutrient transport, phosphorus (P) related *PHOSPHATE TRANSPORTER 1;4* (*PHT1;4*) and *GLYCEROL-3-PHOSPHATE PERMEASE 5* (*G3PP5*) were upwardly mobile in ‘Gala’/‘G202’ at DA (Table S20). On the other hand, N transport-mediating *SLAH3* was downwardly mobile in ‘Gala’/‘M9’ at DA (Table S22).

**Figure 5:**
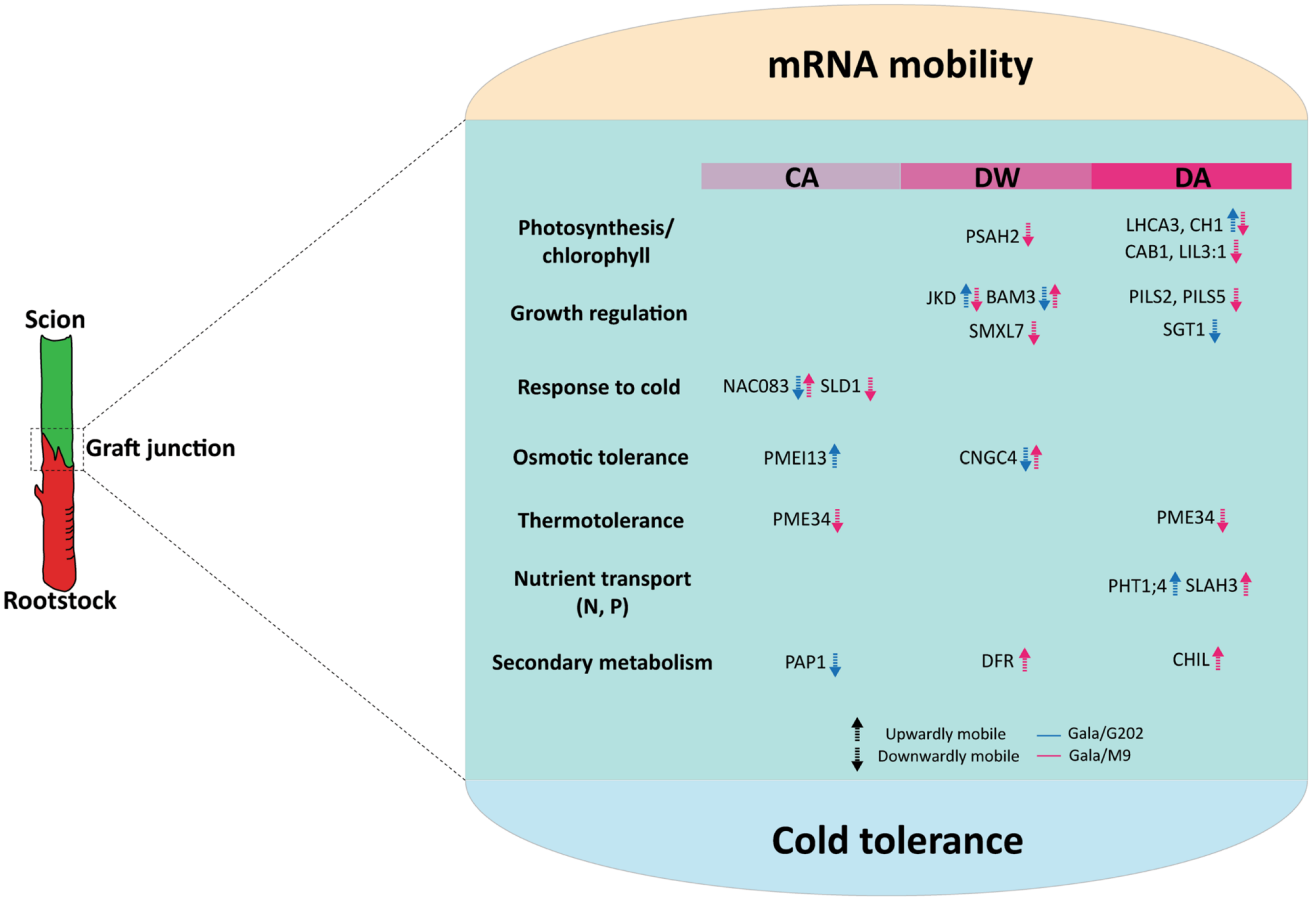
A proposed scheme for mobile mRNA signals associated with cold tolerance in stem vasculature of grafted apple. Candidate mRNA signals that potentially mediate interactive metabolic responses between rootstock and scion are shown with the movement direction. *CA* cold acclimation, *DW* deep winter, *DA* cold de-acclimation.

Summarizing these results, we present the scheme of rootstock-dependent mobile mRNA signals in response to cold in grafted apple (Fig. 5). Several of these have the potential to enhance cold tolerance in grafted apple trees.

### Metabolomics analysis uncovers polyphenol compounds in response to cold

**Figure 6:**
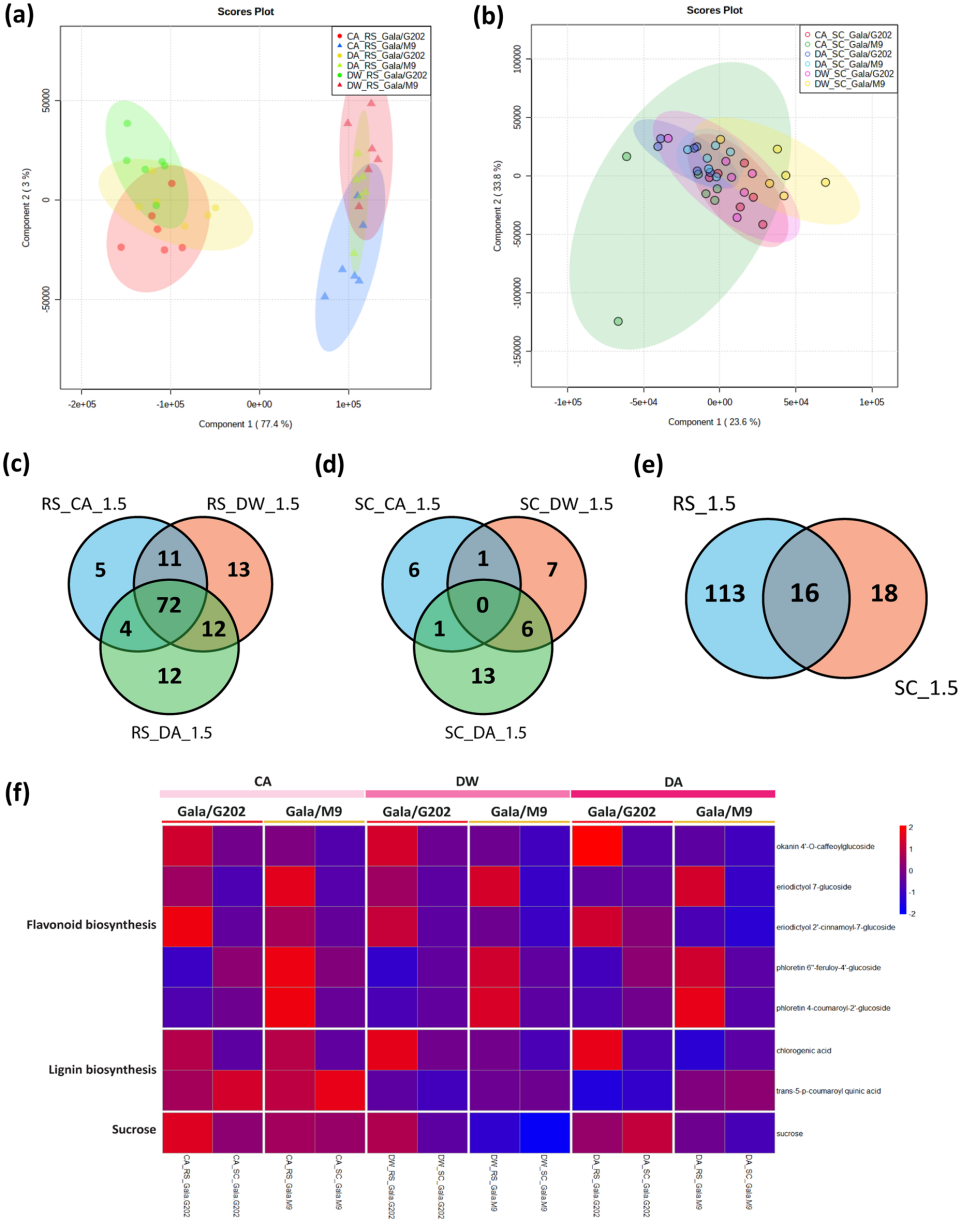

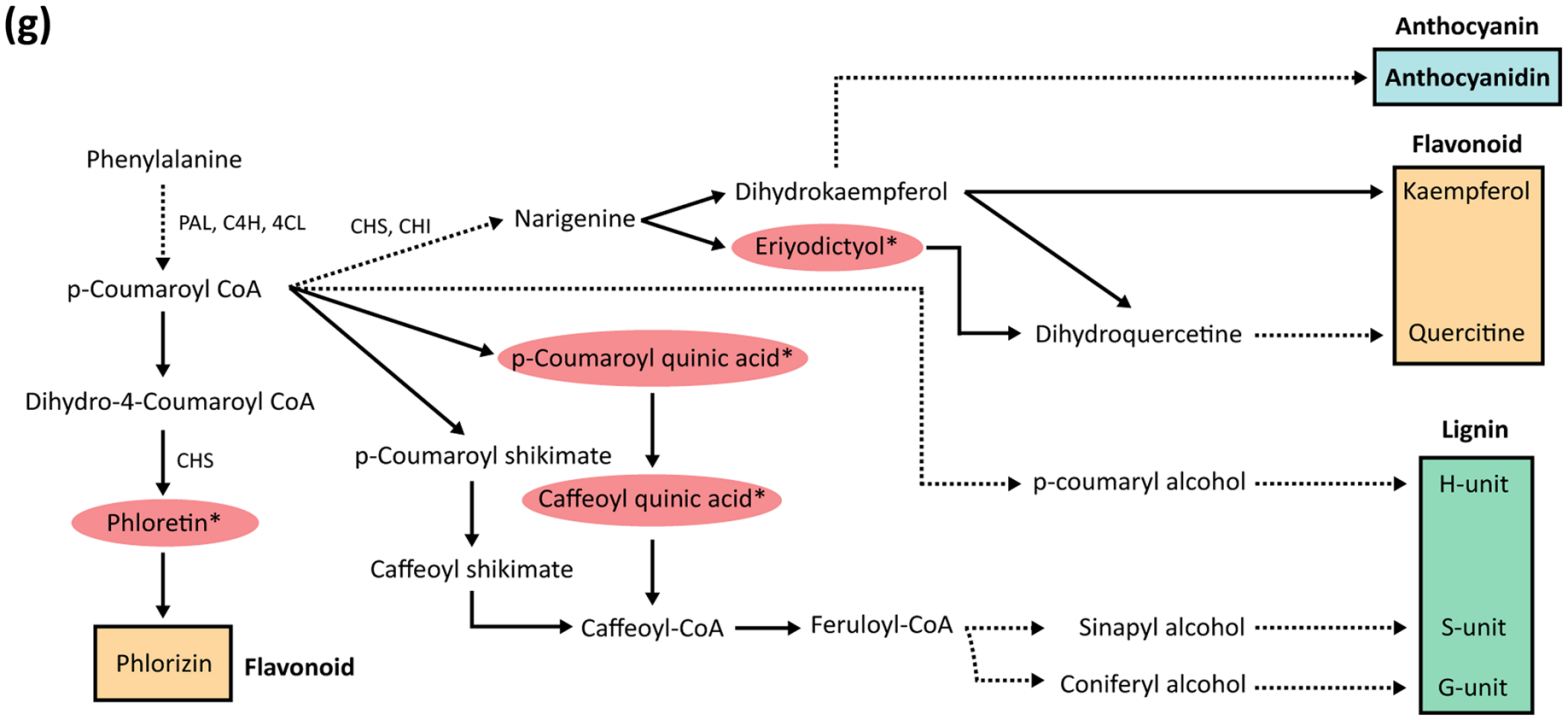
Metabolomics profile using UPLC-QTOF-MS. (**a**,**b**) Principal component analysis and expression pattern of metabolites were analyzed by Partial Least-Squares Discriminant Analysis (PLS-DA). (**a**) rootstock; (**b**) scion. (c-d) Venn diagram of differentially accumulated metabolites across three winter stages (CA, DW, DA) with the criteria of |fold change|≥ 1.5 and p-value ≤ 0.05. (**c**) rootstock; (**d**) scion. (**e**) Venn diagram of the shared metabolites between scion and rootstock tissues in apple graft combinations pooled from all stages. (**f**) Expression profile of eight identified metabolites from the 14 shared scion metabolites. Within the genotype columns, left is rootstock labeled as RS and right is scion labeled as SC. (**g**) Flavonoid biosynthesis pathway in which shared scion metabolites were enriched and potentially considered to be involved. Detected metabolites are marked with asterisks. *CA* cold acclimation, *DW* deep winter, *DA* cold de-acclimation, *RS* rootstock, *SC* scion, *G202* ‘Gala’/‘G202’, *M9* ‘Gala’/‘M9’.

To further provide supporting evidence for the potential mobile signals responsive to cold in grafted apple, we conducted the UPLC-QTOF-MS based-metabolomic analysis of the same stem samples as those used for our RNA-seq (Fig. 6a,b). Across all time three points, 129 and 34 metabolites accumulated (|fold change|≥ 1.5 and *p* ≤ *0.05*) differentially out of 270 and 233 metabolites detected in rootstocks and scions, respectively (Fig. 6c–e, Table S23). We then focused on the 34 scion metabolite set for the chemical identification and examined the patterns of differential levels in metabolic components between two ‘Gala’ scions grafted onto contrasting rootstocks for cold tolerance (Fig. S13, Table S24). Among 16 metabolites shared between rootstocks and scions, eight metabolites identified were compared in rootstock and scion (Fig. 6f). The most abundant metabolites detected were polyphenol compounds that were involved in either flavonoid or lignin biosynthesis pathway (Fig. 6g). Notably, at DA, chlorogenic acid, an intermediate metabolite in lignin biosynthesis was found at a higher level in the scion of ‘Gala’/‘G202’ than in the scion of ‘Gala’/‘M9’ (Fig. S13). The result was in line with our transcriptome analysis, in which lignin biosynthesis seemed to be strongly induced in ‘Gala’/‘G202’ to enhance cold tolerance from mid to late-winter stages (DW and DA) with the upward mobility of relevant genes (*PAL* at DW and *4CL3* at DA) in ‘Gala’/‘G202’ (Fig. S12). In case of flavonoids, we found that at late winter (DA) eriodictyol and phloretin-derived compounds, which participate in the flavonoid biosynthesis pathway as well as the anthocyanin biosynthesis, accumulated more in the scion of ‘Gala’/‘G202’ than in ‘Gala’/‘M9’ (Fig. S13). However, when their levels were compared with those in rootstocks, they showed a contrasting distribution gradient in the two graft combinations (downward in ‘Gala’/‘G202’ and upward in ‘Gala’/‘M9’). These results were partly consistent with the upward mobility of transcripts involved in flavonoid biosynthesis (*CHIL*, *UGT91C1*) in ‘Gala’/‘M9’ at DA stage. Taken together, these metabolomics data support the potential mobile signals associated with seasonal cold tolerance through the vasculature in grafted apple.

## Conclusion

There have been studies about grafted plants to examine the cold-responsive genes or to detect the mobility of signals after grafting^16,50,67^. However, the specific function of signals in mobile mRNAs associated with cold tolerance has remained unknown. Our seasonal transcriptome profiling of stems in two apple graft combinations reveals comprehensive information about the expression dynamics of genes in response to cold. By exploring the correlated expression patterns of DEGs that were shared by scion and rootstock across cold stages, we characterized the 544 potentially mobile mRNAs moving up or downward through the vasculature, which might contribute to the differential responses to cold in grafted apple. Our genome-wide variant screening analysis and functional assays further validated the mobility of mRNAs that might control the rootstocks' influence on scions' cold tolerance, implying the exchange of mRNAs along the graft junction in association with seasonal cold stress conditions.

One main reason for developing a better understanding of cold tolerance is to apply this knowledge to orchard management of grafted tree crops with improved tolerance to external changes in temperature. We propose mobile mRNA signals for seasonal cold tolerance based on these investigations. These include genes involved in photosynthesis/chlorophyll (*LHCA3*, *PSAH2*, *CAB1*), growth regulation (*JKD*, *BAM3*, *SGT1*), response to cold (*VNI2*, *SLD1*), osmotic (*PMEI13*, *CNGC4*) and thermo- (*PME34*) tolerance, nutrient transport (*PHT1;4*, *SLAH3*), and secondary metabolism (*PAP1*, *DFR*, *CHIL*). The detailed understanding of mRNA movement associated with cold tolerance will provide a comprehensive insight into signal exchanges between rootstocks and scions in response to cold and serve for future research to enhance cold tolerance in apple.

## Supplementary Information

Supplementary Figures.

Supplementary Information 1.

Supplementary Tables.

## Data Availability

All sequencing data were deposited in the National Center for Biotechnology Information Sequence Read Archive database bearing the BioProject ID PRJNA716741.
