# Supplementary material for: Identification of genes associated with the regulation of cold tolerance and the RNA movement in the grafted apple
Source: Sci Rep. 2023 Jul 18;13:11583. doi: 10.1038/s41598-023-38571-2 (PMC10354032; doi:10.1038/s41598-023-38571-2)
Supplement: Supplementary file 1 — Supplementary Figures. [file 41598_2023_38571_MOESM1_ESM.docx]

**Supporting Information Figure S1-S13**


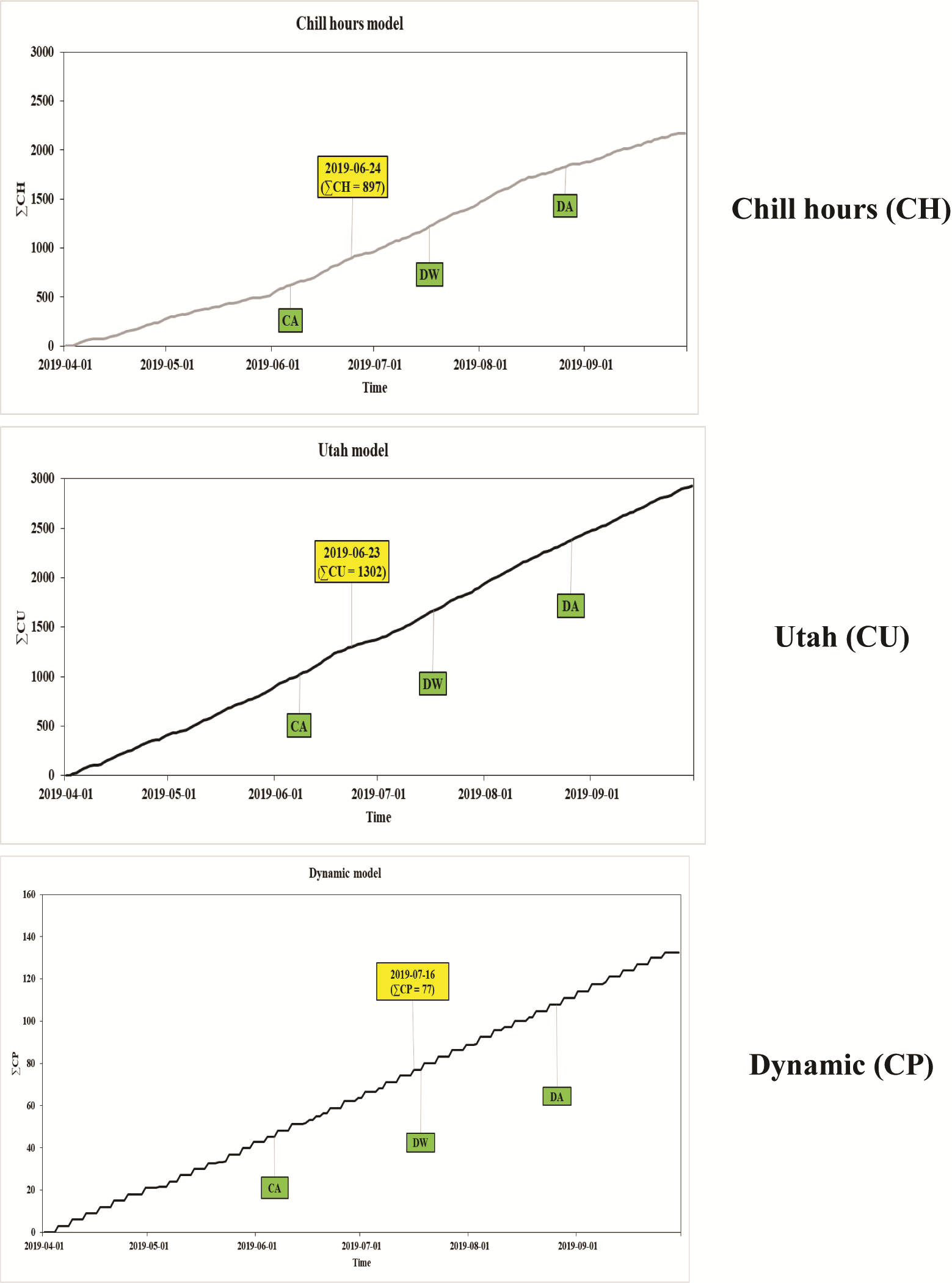


**Figure S1.** Cumulative chill units at three winter stages: CA (early winter/cold acclimation, 6^th^ June), DW (deep winter, 17^th^ July), DA (late winter/cold de-acclimation, 26^th^ August). The reference chilling requirement for ‘Gala’ genotype used for the estimation of endodormancy release were 908 chill hours (CH), 1307 chill units (CU) and 77 chill portions (CP).

**
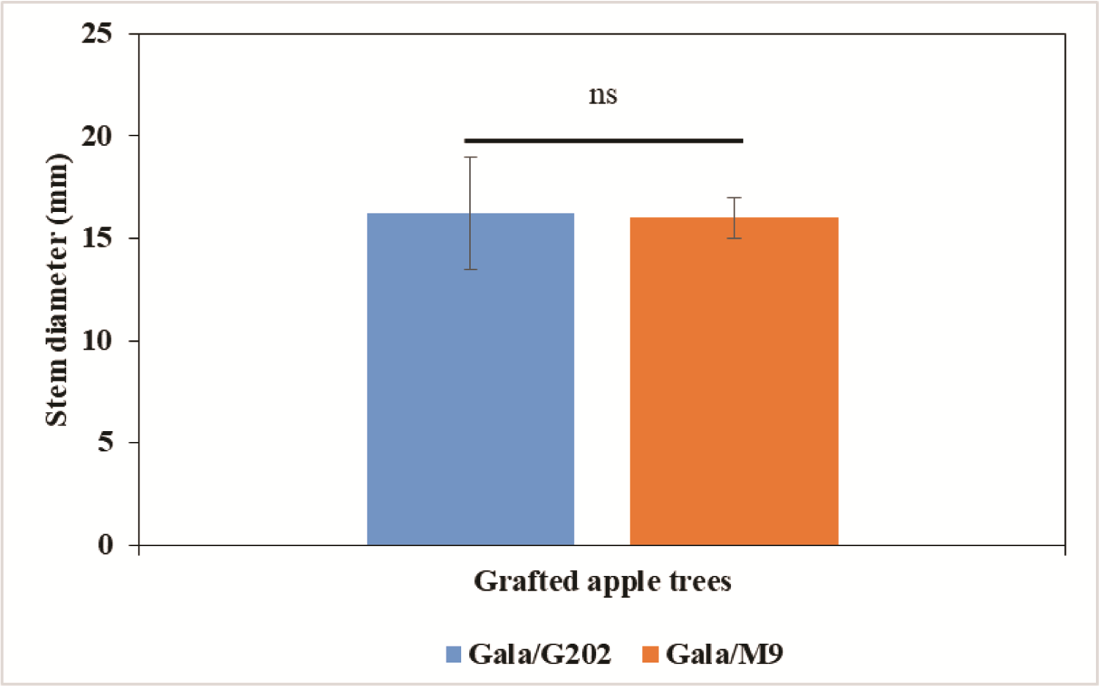
**

**Figure S2.** Stem diameter of ‘Gala’/‘G202’ and ‘Gala’/‘M9’. There was no significant difference between two apple graft combinations.


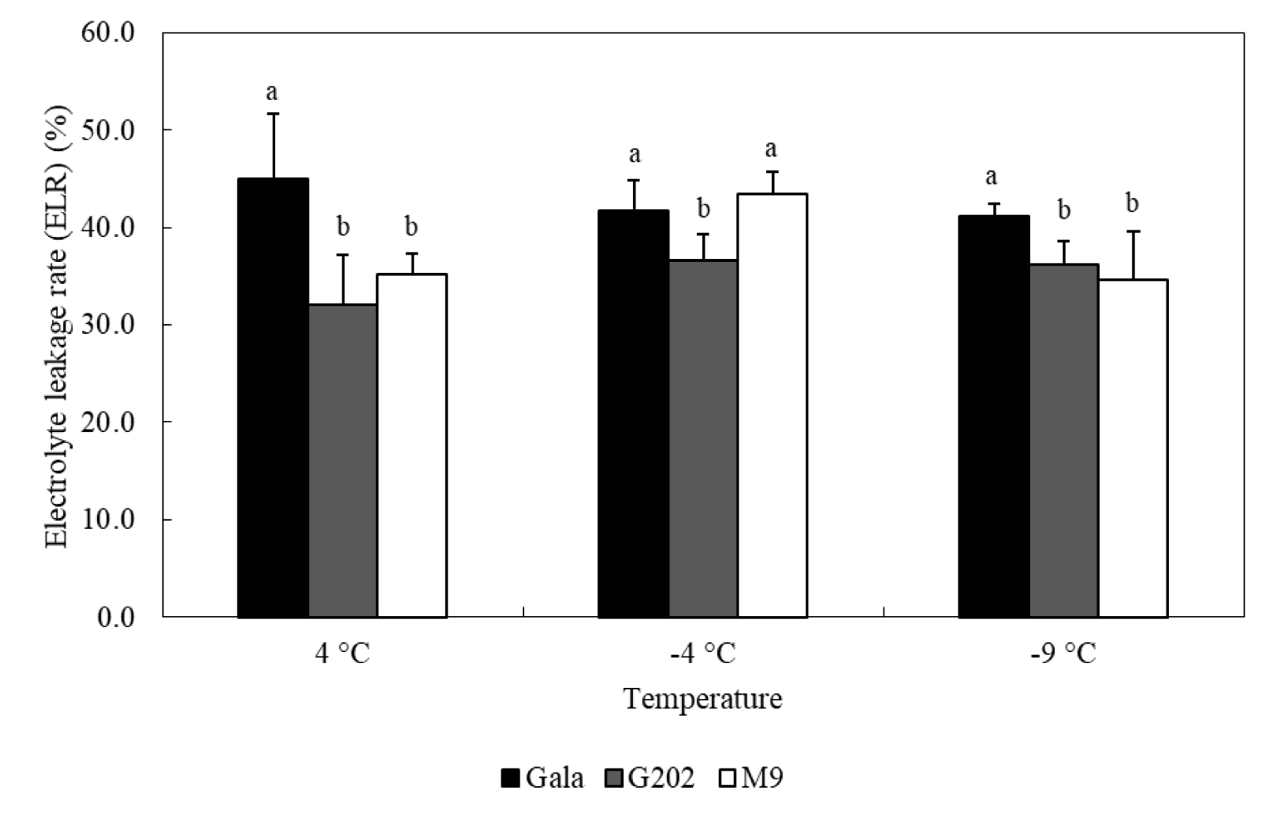


**Figure S3.** Electrolyte leakage rate (ELR) among three genotypes (‘Gala’, ‘G202’, ‘M9’) measured at three chill/freezing temperatures: 4, -4, -9 °C.


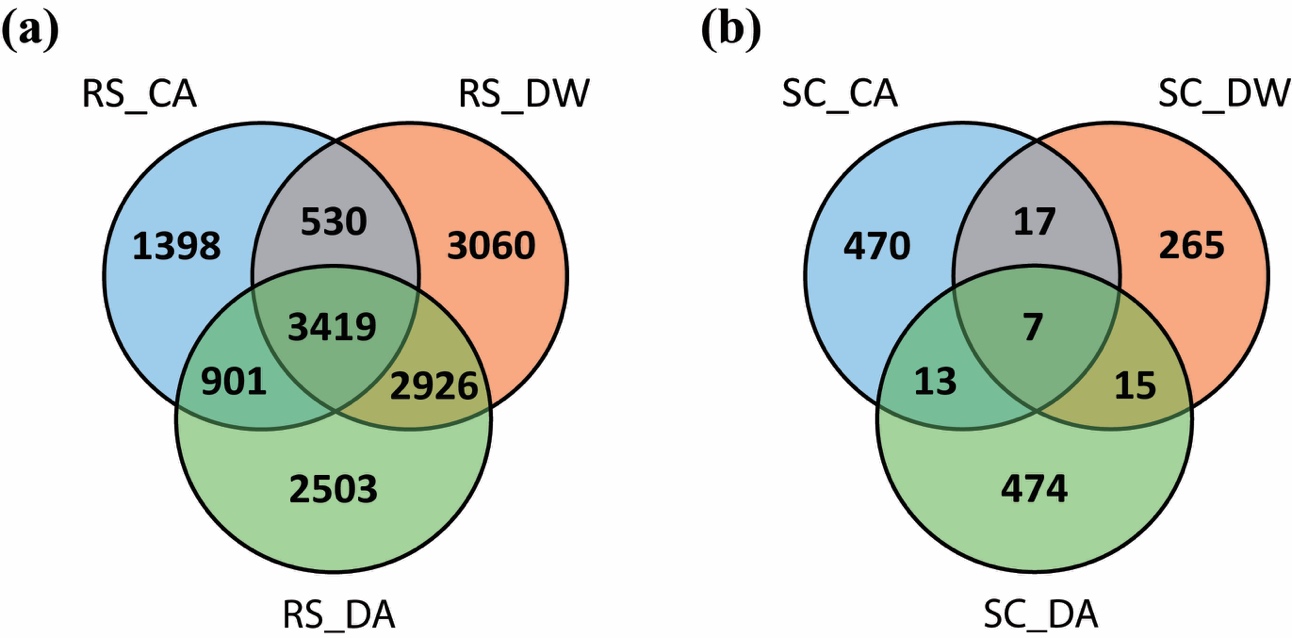


**Figure S4.** Venn diagram of DEGs detected with a criteria of |fold change| ≥ 1.5 and FDR < 0.05. (a) rootstock; (b) scion. Abbreviations: RS, rootstock; SC, scion; CA, cold acclimation; DW, deep winter; DA, cold de-acclimation.


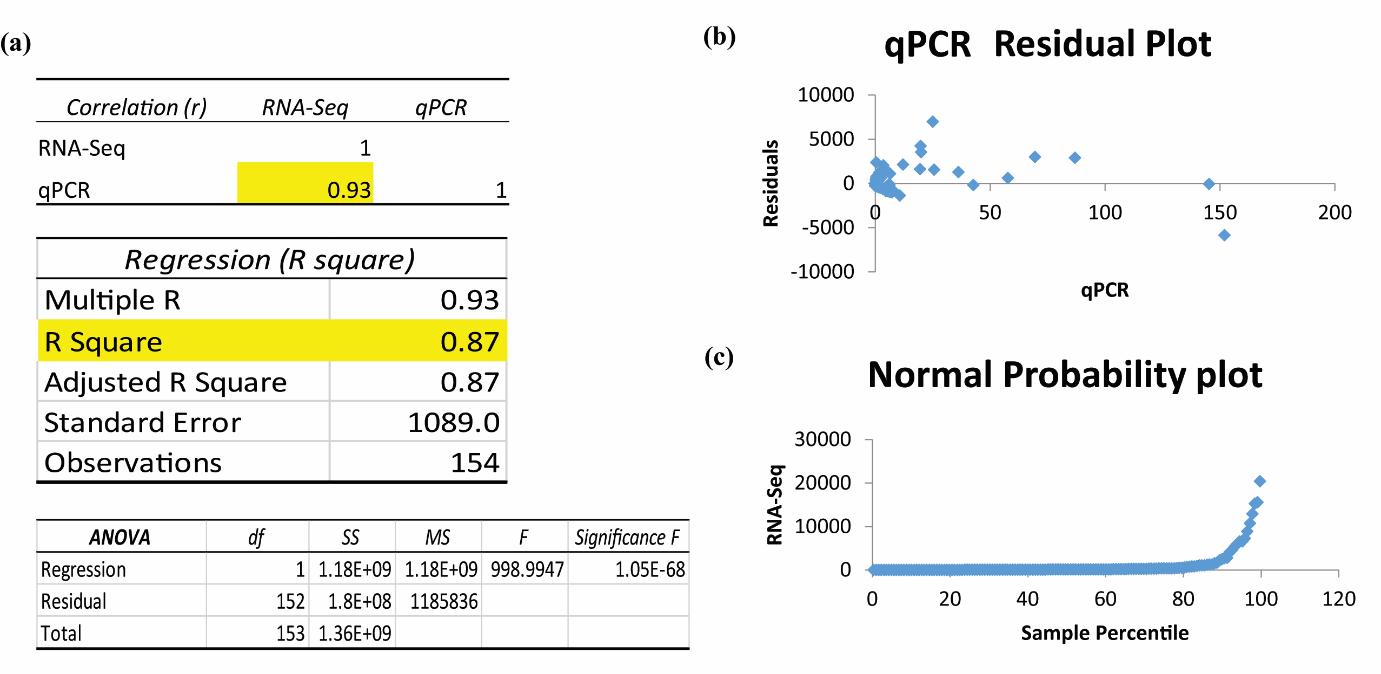


**Figure S5.** qPCR validation for RNA-seq. (a) correlation and regression analyses were conducted between data of RPKM and qPCR with a set of 13 selected genes. (b) qPCR residual plot. (c) normal probability plot.


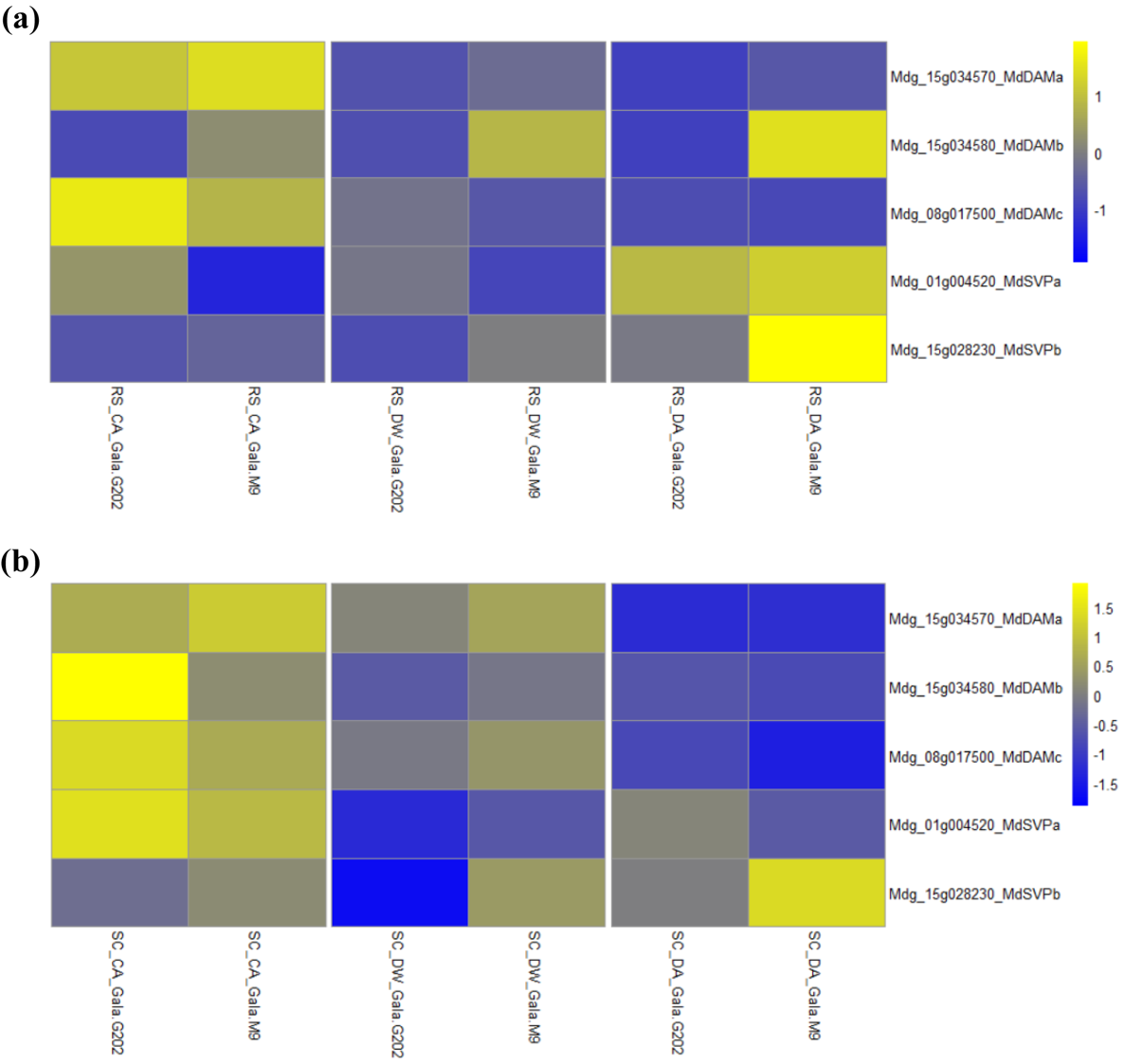


**Figure S6.** Expression heatmaps of dormancy-related genes (*MdDAMs*, *MdSVPs*). (a) rootstock; (b) scion. Abbreviations: RS, rootstock; SC, scion; CA, cold acclimation; DW, deep winter; DA, cold de-acclimation.

**
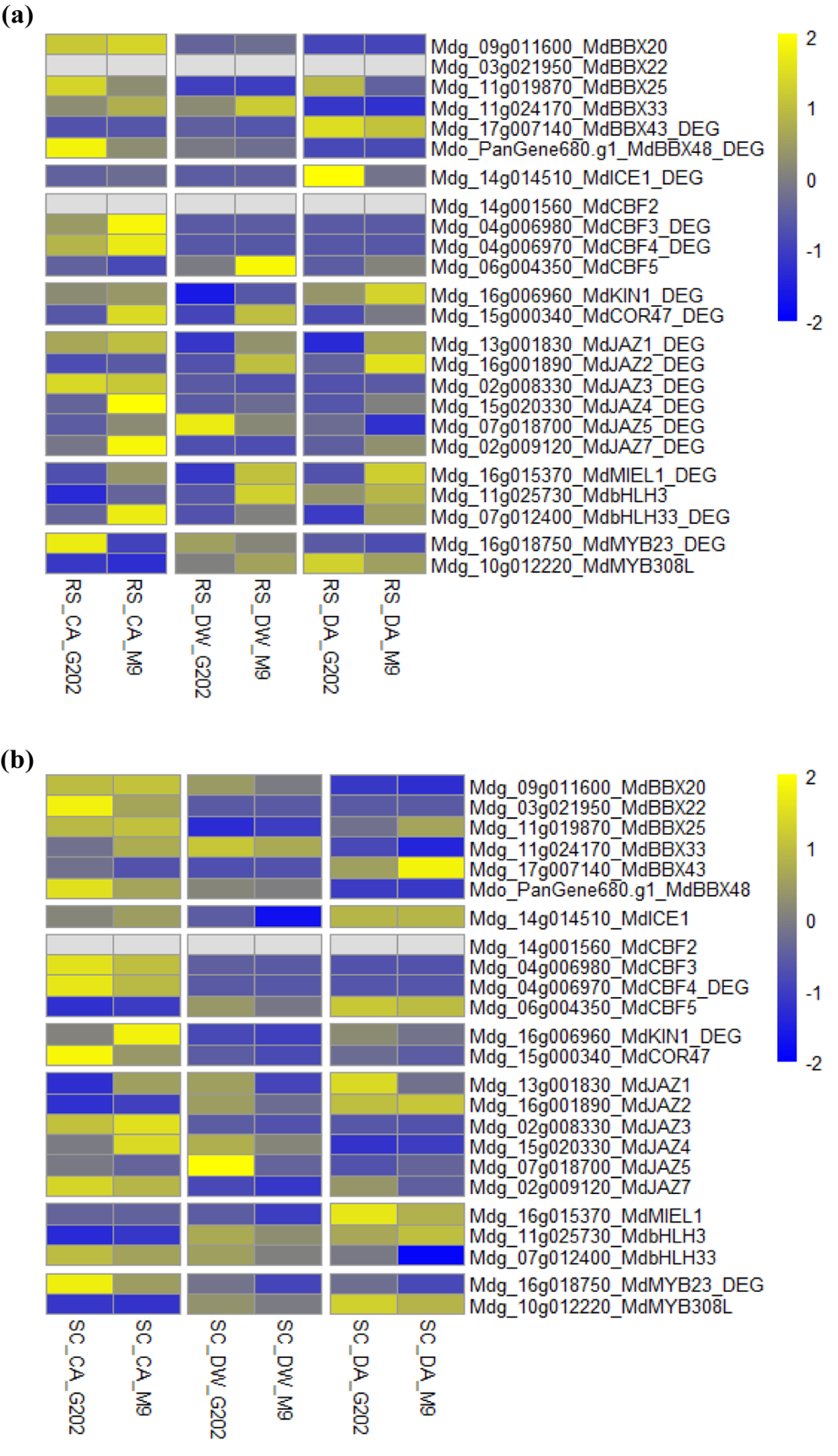
**

**Figure S7.** Expression profile of genes involved in ICE-CBF cold tolerance pathway mediated by JA signaling conserved in apple (An et al., 2021). (a) rootstock; (b) scion. Abbreviations: RS, rootstock; SC, scion; CA, cold acclimation; DW, deep winter; DA, cold de-acclimation; G202, ‘Gala’/‘G202’; M9, ‘Gala’/‘M9’.

**
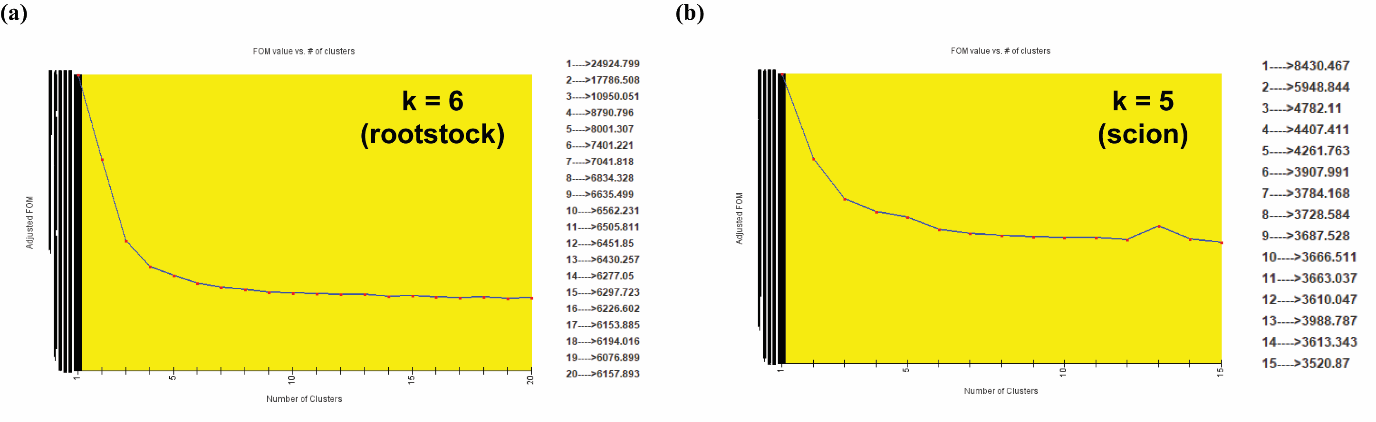
**

**Figure S8.** Figure of merit (FOM) analysis. (a) 14,747 rootstock DEGs; (b) 1,261 scion DEGs.

**
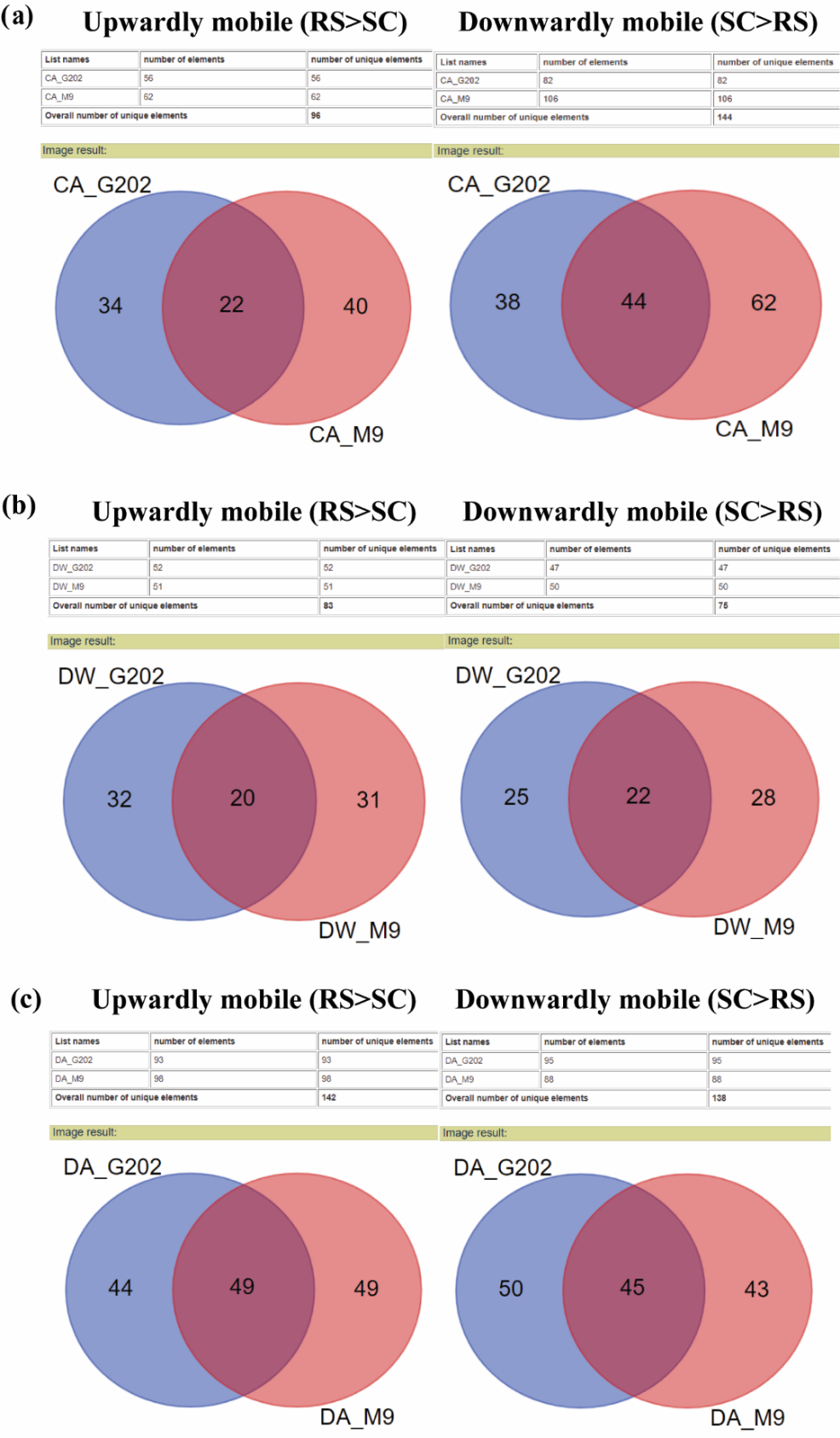
**

**Figure S9.** Venn diagrams of seasonal flow DEGs at three winter stages categorized by the potential mobility direction: (a) CA; (b) DW; (c) DA. Abbreviations: RS, rootstock; SC, scion; CA, cold acclimation; DW, deep winter; DA, cold de-acclimation; G202, ‘Gala’/‘G202’; M9, ‘Gala’/‘M9’.


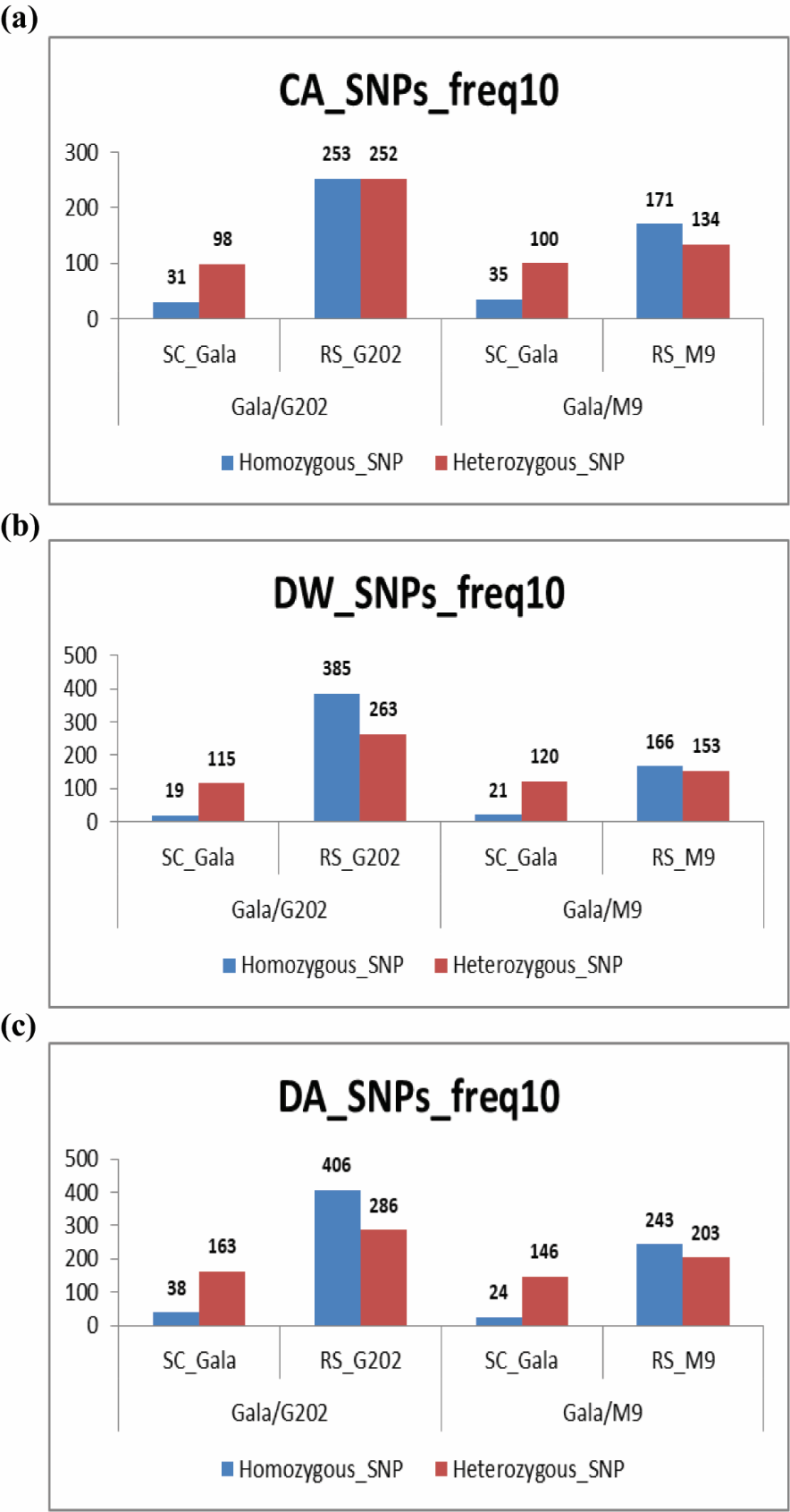


**Figure S10.** Homozygous and heterozygous SNPs detected from 544 DEGs at three winter stages (CA, DW, DA) with the parameters of coverage 10, count 10, and frequency 20%. (a) CA; (b) DW; (c) DA. Abbreviations: RS, rootstock; SC, scion; CA, cold acclimation; DW, deep winter; DA, cold de-acclimation.


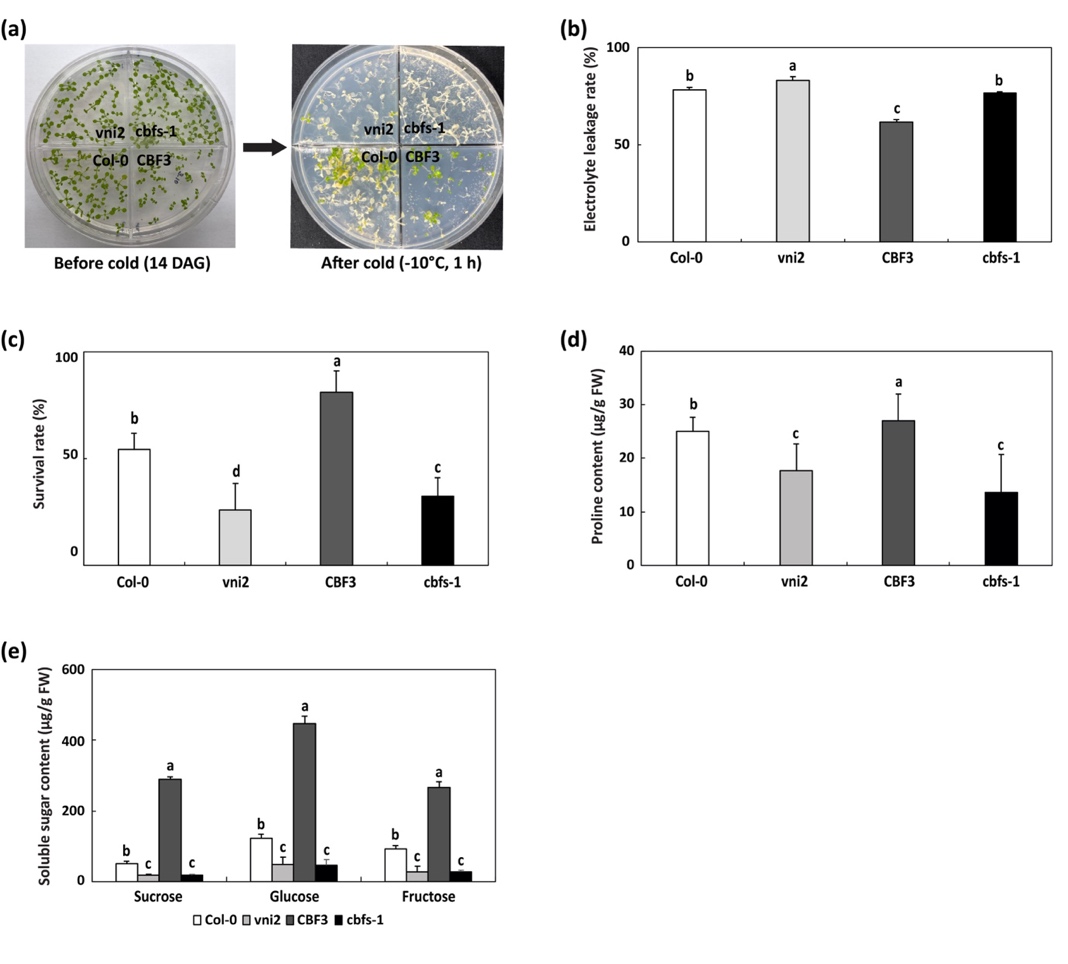


**Figure S11.** **Comparison of cold tolerance phenotypes among *Arabidopsis* genotypes. (a-c)** The freezing assay of four genotypes (Col-0, *vni2*, *CBF3*, *cbfs-1*). Fourteen-day-old seedlings grown on medium at 25ºC were treated in a freezing chamber from 0ºC and the temperature was lowered at a rate of 1 °C/h followed by cold acclimation at 4°C. The seedlings were kept in the chamber until −10°C for 1 h. **(a)** Photographs were taken after 3 d of recovery at 25°C. **(b)** Electrolyte leakage rate (ELR) and **(c)** the survival rate were examined. There were six independent replicates for ELR analysis (n=10, pooled) and there three independent replicates for counting the survival rate (n=35, pooled). **(d-e)** The chilling assay of four genotypes. Fourteen-day-old seedlings grown on medium at 25ºC were treated at 4°C for 3 d. The content of proline **(d)** and sugars (sucrose, glucose and fructose) **(e)** were measured. There were four independent replicates (n=3, pooled) for the measurement of proline and soluble sugars. Line on the bars show SE of the mean and different letters indicate significant difference by Duncan’s multiple range test (DMRT) (*p* < 0.05).

**
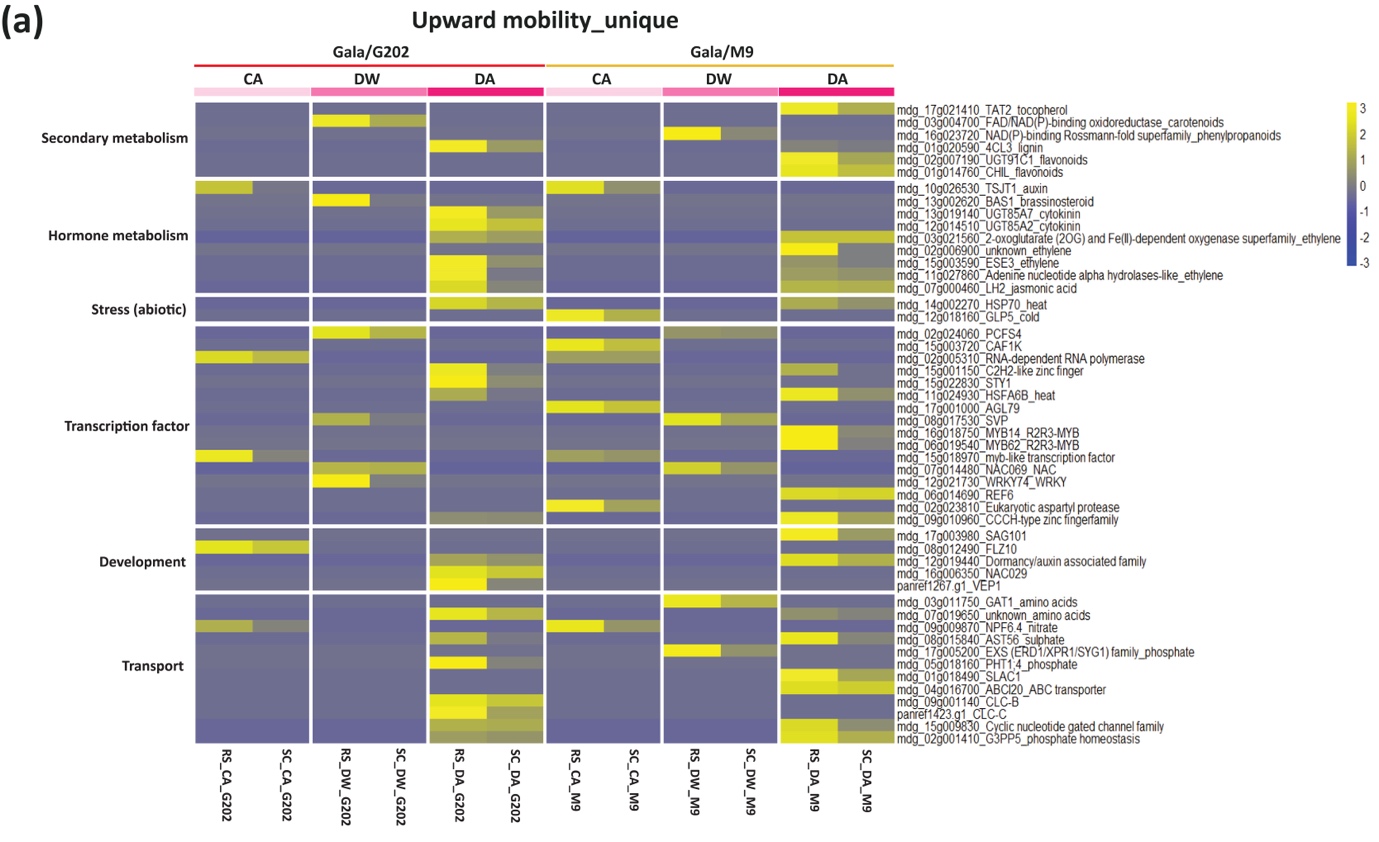
**

**
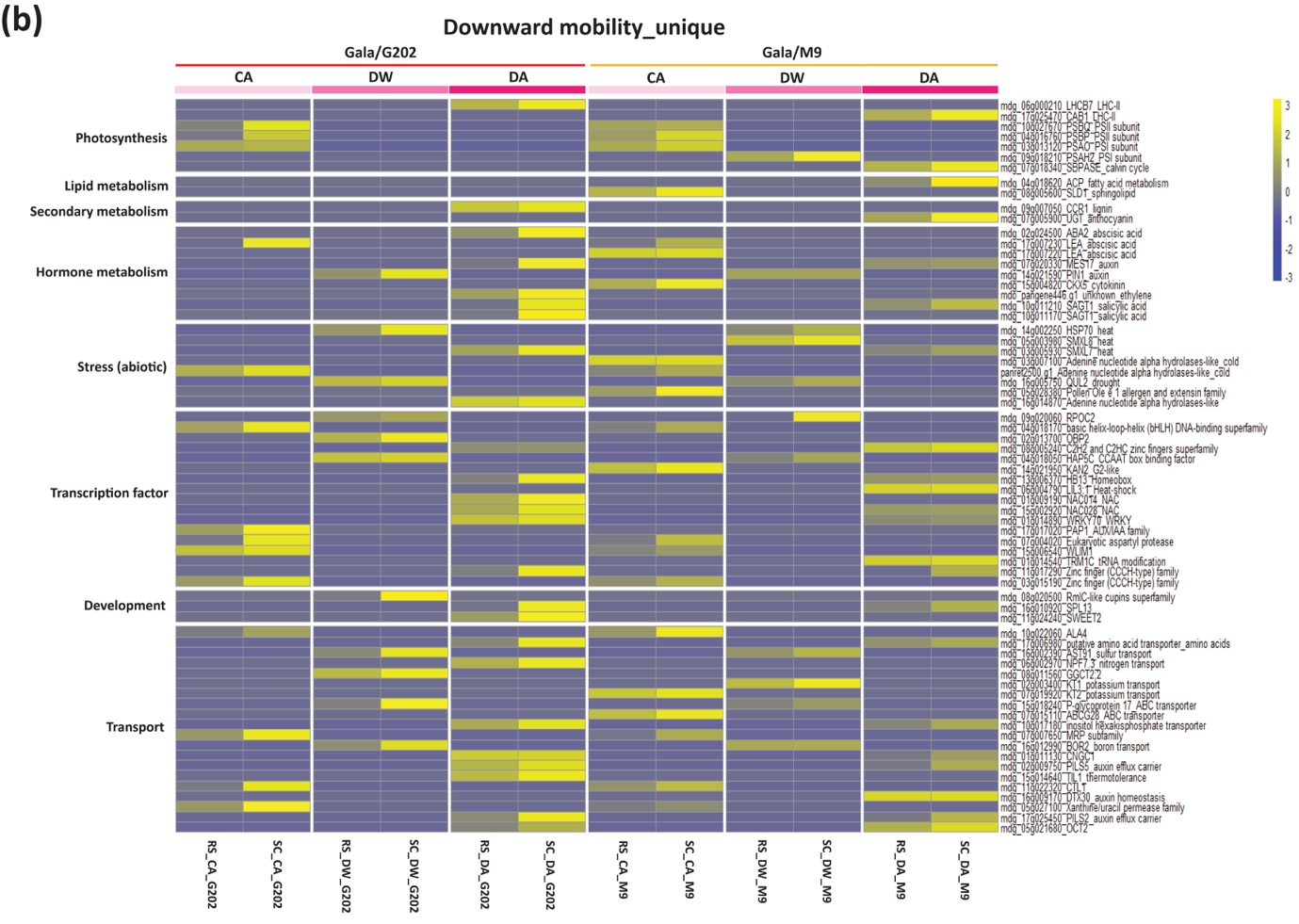
**

**
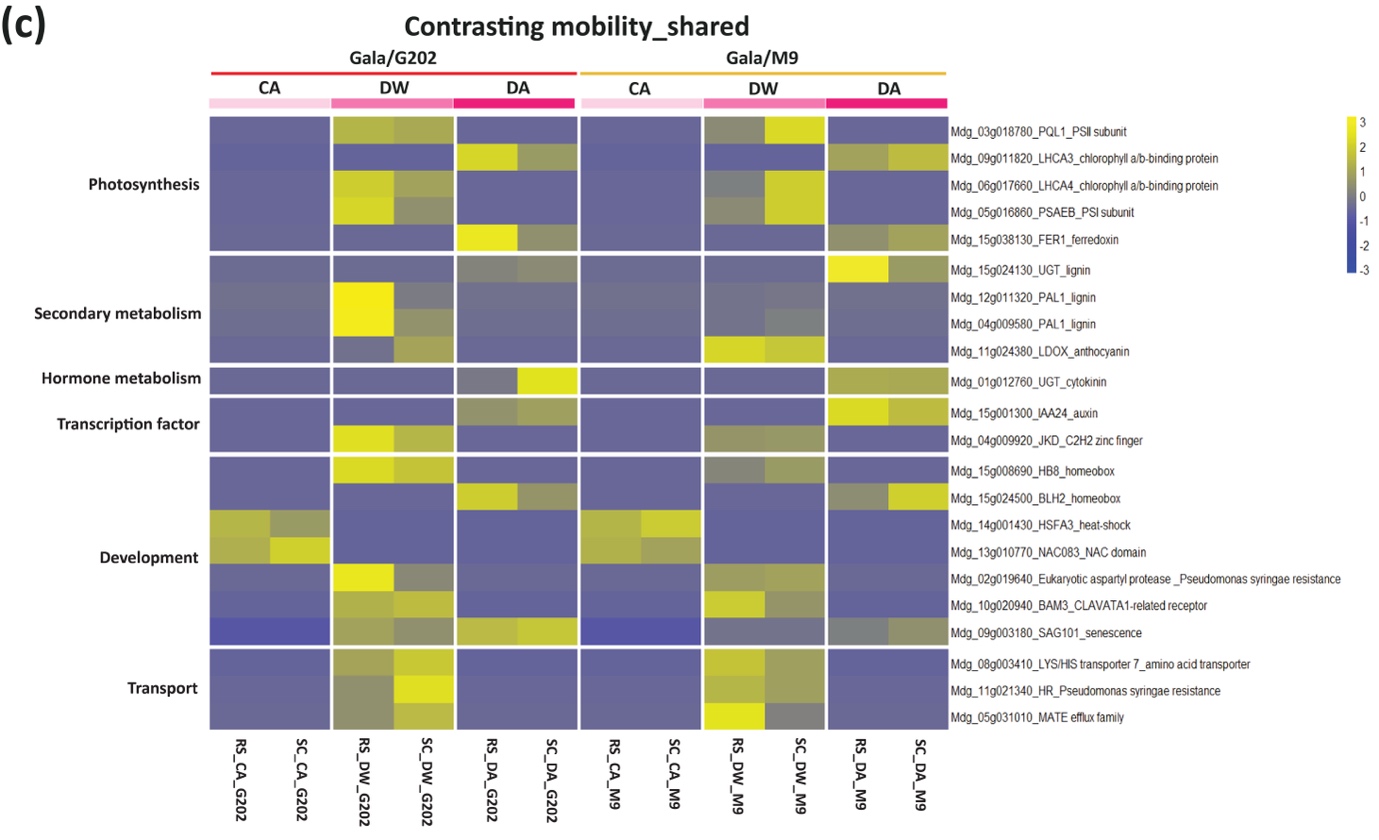
**

**Figure S12.** Expression heatmaps of seasonal flow DEGs unique to mobility direction among eight main functional categories. (a) upward mobility; (b) downward mobility; (c) contrasting mobility. Abbreviations: RS, rootstock; SC, scion; CA, cold acclimation; DW, deep winter; DA, cold de-acclimation; G202, ‘Gala’/‘G202’; M9, ‘Gala’/‘M9’.


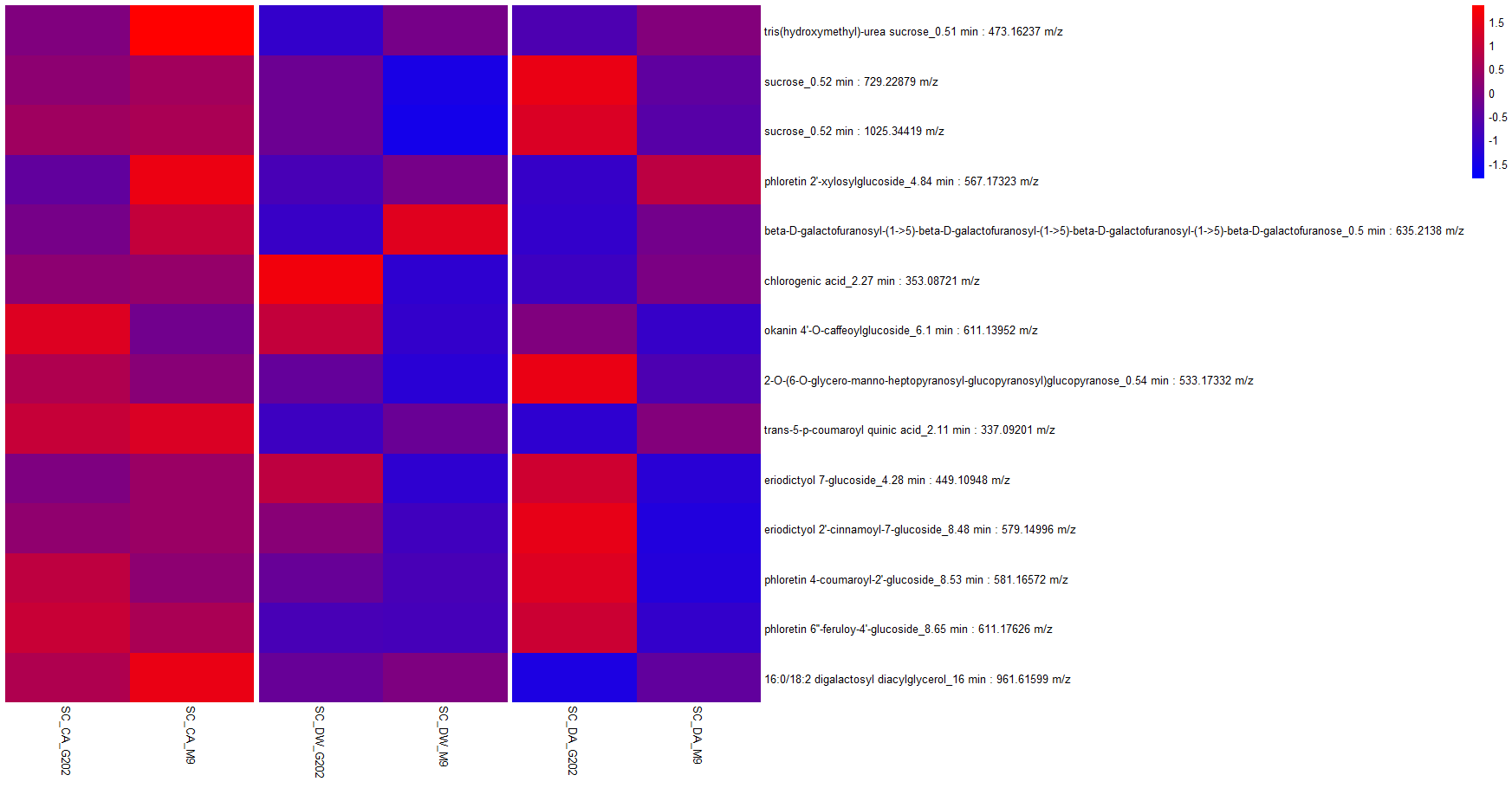


**Figure S13.** Expression heatmap of 14 ‘Gala’ scion metabolites grafted onto two contrasting rootstocks (‘G202’, ‘M9’) collected at winter stages detected by UPLC-QTOF-MS with chemical identification. Abbreviations: RS, rootstock; SC, scion; CA, cold acclimation; DW, deep winter; DA, cold de-acclimation; G202, ‘Gala’/‘G202’; M9, ‘Gala’/‘M9’.
