# Supplementary material for: Identification of genes associated with the regulation of cold tolerance and the RNA movement in the grafted apple
Source: Sci Rep. 2023 Jul 18;13:11583. doi: 10.1038/s41598-023-38571-2 (PMC10354032; doi:10.1038/s41598-023-38571-2)
Supplement: Supplementary file 2 — Supplementary Information 1. [file 41598_2023_38571_MOESM2_ESM.docx]

**Identification of genes associated with the regulation of cold tolerance and the RNA movement in grafted apple**

Youngsuk Lee, Nam V. Hoang, Van Giap Do, Toshi M. Foster, Tony K. McGhie, Seonae Kim, Sang Jin Yang, Ju-Hyeon Park, Jongsung Park, and Ji-Young Lee

Supporting Information Methods S1-S4

Supporting Information Notes S1-S5

Supporting Information Methods S1-S4

Method S1. Estimation of the expected dormancy of ‘Gala’ genotype.

The expected time of endodormancy release for scion ‘Gala’ genotype was calculated based on various chill requirement (CR) satisfaction models including chill hours (CH), chill units (CU) and chill portions (CP) ^1-4^. CRs required for dormancy release of ‘Gala’ are known as 77 CP, 1307 CU and 908 CH as previously studied ^5^. By calculating each chill unit converted from hourly temperature data at Clyde site, the time that cumulative chill units exceed CR was between late June and mid July (**Table S1**), suggesting that the transition from endo- to eco-dormancy occurred between first two time points CA and DW of our sample collection (**Fig. S1**).

Method S2. RNA and DNA sequencing, quantitative reverse transcription PCR and single nucleotide polymorphism (SNP) detection.

RNA-seq read processing, mapping and expression data analysis

Raw reads were trimmed to remove low quality and short sequences. Only PE reads with a minimum quality score of 0.05 (equivalent to Phred Q13), ≤2 of ambiguous nucleotides, and a length of ≥75 bp were retained for further analyses. Trimmed reads were aligned to the apple pan-genome reference ‘Gala’ haploid ^6^ which contains 49,944 protein coding genes by RNA-seq Analysis tool in CLC genomics workbench 21 (QIAGEN, Hilden, Germany) using the parameters of both minimum length fraction and minimum similarity to 0.8 (**Table S5**). Raw read counts were normalized to reads per kilobase per million (RPKM) for each gene. Baggerley’s test was applied to compared for differentially expressed genes (DEGs) with the statistics analysis as described in ^7^. DEGs were determined by comparing between the scion and rootstock samples of the two genotypes at three timepoints with a |fold change| ≥1.5 and a false discovery rate (FDR) value of ≤0.05. DEG clusters were analysed using K-means method in MeV software ver4.9.0 ^8^. GO term enrichments were investigated using agriGO enrichment tool ^9^ using gene IDs from the apple GDDH13 V1.1 genome ^10^. KEGG pathway enrichments were explored using DAVID Web tool ^11,12^.

Quantitative reverse transcription PCR (qRT-PCR)

First-strand complementary DNA was synthesized using 1 μg of total RNA, oligo dT primer, and Transcriptor Reverse Transcriptase (Roche, Penzberg, Germany). qRT-PCR analysis was performed in LightCycler 480 SYBR Green I Master mix (Roche, Germany) on a Roche 480 LightCycler (Basel, Switzerland). Complementary DNA (20-fold dilution) was used as a template (5 μL) in a 20 μL reaction volume. For each sample type, there were four to six technical replicates. PCR cycles were as follows: initial denaturation at 95°C for 5 min, followed by 45 cycles of 95°C for 10 s, 65°C for 15 s, and 72°C for 12 s, and a final melt curve analysis to determine the amplification of a single product. Primers were designed using Primer-Blast (http://www.ncbi.nlm.nih.gov/tools/Primer-Blast/) to span an intron if possible, with 100-150 bp product size (**Table S4**). MDP0000336547 (SGF29 tudor-like domain) was selected as the reference gene ^13^. Primer efficiencies and the relative expression levels of targets were calculated using the Roche 480 Light Cycler software E-Method ^14^.

Whole-genome resequencing (WGS) of ‘Gala’, ‘G202’, and ‘M9’ genotypes

Genomic DNA was extracted from branch tissues of three genotypes (‘Gala’, ‘G202’, ‘M9’) using a modified CTAB method. DNA libraries were assessed using Agilent 4200 Tapestation (Santa Clara, CA, USA) and constructed using an Illumina Novaseq platform (San Diego, CA, USA) with 2×151-bp paired-end (PE) reads. Trimmed reads with a quality score of <0.05 (equivalent to Phred Q13), ≤2 of ambiguous nucleotides, and a length of ≥75 bp were aligned to the same reference (pan-genome ‘Gala’ haploid) and same mapping parameters as RNA-seq (similarity 0.8, length fraction 0.8) using Map Reads to Reference tool in CLC genomics workbench. The average depth of coverage were 23x, 30x and 40x for ‘Gala’, ‘G202’ and ‘M9’ respectively, while the percentage of mapped reads ranged from 93.9 to 95.6 % (**Table S3**). Genome heterozygosity of each genotype was determined by Genomescope 2.0 ^15^.

Single nucleotide polymorphism (SNP) detection

To facilitate the detection of mobile mRNA signals that were exchanged between rootstock and scion in the two graft combinations, we conducted SNP detection using WGS and whole transcriptome sequencing data. SNPs were then filtered with different minimum allele frequency (MAF) cutoffs >20%, 20-80% and 100% (both minimum coverage and count set to 10) for total, heterozygous and homozygous SNPs, respectively. The genotype-unique SNPs associated with the cold-tolerant ‘G202’ and cold-susceptible ‘M9’ (that were not detected in the ‘Gala’ data) were used to validate rootstock-derived candidate mobile mRNAs influencing cold tolerance in scion (‘Gala’) in RNA-seq data. Heterozygous and homozygous RNA-seq SNPs were determined by a MAF at 10-75% and 100%, respectively. For a given rootstock genotype-unique SNP determined by WGS data analysis, it was first confirmed with the RNA-seq data of the corresponding rootstock (e.g., to be homozygous in both cases), then checked in the scion sample to infer whether the mRNA associated with it was mobile or not. A MAF cutoff of 10% and minimum coverage and count of 10 were used for SNP detection in RNA-seq data. Based on the relative abundance of SNP frequency present in the RNA-seq data between scions and rootstocks, the direction of RNA movement was determined for each SNP-associated gene with the annotation of mobility confidence levels (0-3) by comparing the consistency of genotypic origin, cold stage and the original direction of potential mRNA mobility with the result of seasonal flow DEG analysis (see **Tables S17**).

Quantification and statistical analyses

The heatmap of genes showing the hierarchical clustering was analyzed using the Pheatmap R package (<https://cran.r-project.org/web/packages/pheatmap/>). Functional annotations were generated using online Mercator software tool ^16^ to predict putative Arabidopsis orthologs in apple transcripttome data. Other statistical analyses were performed in Microsoft Excel 2016 and R program (<http://www.R-project.org/>).

Method S3. Agroinfiltration of *MdTSJT1* into tobacco for transient overexpression assay.

For infiltration, EHA105 cells were inoculated in Luria-Bertani (LB) medium and cultured at 28°C with 200 rpm for at least 24 h. Cells were resuspended in an infiltration buffer and infiltrated into the leaf of mature tobacco plant (40 days after sowing) using needless syringes. Only one leaf per plant was injected with each construct for transient overexpression assay. The transformed tobacco plants were incubated at 25°C under 16 h light/8 h dark photoperiod condition for 3 d and then placed into Conviron PGC-20 growth chamber (Winnipeg, Canada) for a short term cold treatment. The temperature was lowered to 4°C with a rate of -5°C/h for cold acclimation (CA, early), maintained at 4°C for 7 d reflecting cold tolerance (DW, deep) and then increased to 25°C with a rate of 5°C/h representing a stage of de-acclimation to cold (DA, late). Samples were collected from different tissues: the infiltrated leaf, petiole and adjacent stem above/below the infiltrated leaf. Samples were collected from four different time points including before the cold treatment (3 d after the infiltration; $T_{1}$) and three cold stages: CA (early; $T_{2}$), DW (deep; $T_{3}$) and DA (late; $T_{4}$). There were six to eight biological replicates. Relative expression levels of transcripts were analyzed by qRT-PCR.

Method S4. Cold phenotypic measurements of *Arabidopsis* genotypes and grafting experiment

**Soluble sugars measurement**

The 14-day-old *Arabidopsis* seedlings grown on medium at 25°C were treated at 4°C for 3 d of cold stress. Soluble sugar content was measured using the Dionex Ultimate 3000 High Performance Liquid Chromatography (HPLC) system (Thermo Scientific, Sunnyvale, CA, USA) with RI-101 detector (Shodex, Tokyo, Japan). Each 0.1g ground sample was dissolved in 1mL of distilled water and boiled for 1 h at 97°C. After cooling down at room temperature, samples were filtered through 0.22-µm micropore membrane and analyzed with a 300 x 6.5 mm Sugar-pak column (Waters, Milford, MA, USA) with distilled-deionized water as a mobile phase for the separation. The injection volume of each sample was 10 μL and the flow rate was 0.5 mL/min.

**Proline content measurement**

The 14-day-old *Arabidopsis* seedlings grown on medium at 25°C were treated at 4°C for 3 d of cold stress. Proline content was measured using a modified ninhydrin method as previously reported ^17^. Each 0.1 g ground sample was mixed with 1 mL of 3% sulfosalicylic acid and centrifuged at 13,000 rpm for 10 min at 4°C. Supernatants were mixed with acetic acid and acidic ninhydrin solution and then boiled in a water bath for 30 min. After cooling down on ice, the proline content was measured at 520 nm of spectrophotometric absorbance using the CLARIOstar microplate reader (BMG Labtech, Ortenburg, Germany). The standard curve was made with a range of L-proline (0-50 µg) and the concentration of sample was calculated.

**Freezing tolerance assay and electrolyte leakage rate (ELR)**

To assess freezing tolerance, 14-day-old *Arabidopsis* seedlings grown on medium at 25°C were treated in a freezing chamber from 0°C and the temperature was lowered to −10°C at a rate of 1 °C/h followed by cold acclimation treatment at 4°C. After freezing treatment, the plants were put at 4°C in the dark for 12 h and then transferred to 25°C for an additional 3 d. The survival rate (35 seedlings for each genotype) was counted. Photographs were taken after 3 d of recovery at 25°C. Results were similar in three independent experiments. For the ELR measurement, ten individual seedlings of 14-day-old *Arabidopsis* seedlings grown on medium at 25°C were pooled and put in a closed sample tube containing 100 μL of distilled water and placed onto a programmable thermo-controller DS-8504-M-S for cold treatment (Daewon Science, Seoul, Korea). Six biological replicates were collected for this analysis. Samples were treated with successive cold temperatures from 0 to −10°C lowered at a rate of 1°C/h followed by cold acclimation treatment at 4°C for 3 h and collected at the designated temperatures. After thawing gradually, samples were transferred to a new tube containing 15 mL of distilled water and placed onto a shaking incubator at 24°C and 150r/min for 24 h. After the incubation, the initial and maximal EC were measured to calculate ELR.

***Arabidopsis* grafting experiment**

Seeds of *Arabidopsis thaliana* wile-type Col-0 and *vni2* mutant sterilized in 70% ethanol were germinated on plates containing ½ MS agar media supplemented with 1% sucrose and 1% agar by vertically incubating for 6 d at 22 °C under the 16 h light/8 h dark photoperiod conditions. The hypocotyls of seedlings were cut with a sterile razor blade under Leica S6E dissecting microscope (Leica Microsystems, Germany) and wild-type or mutant scion shoots were placed carefully on top of mutant or wild-type rootstock roots to make different graft combinations. After grafting, the re-connected seedlings were recovered in sealed ½ MS agar media without sucrose, by vertically incubating at 22 °C under the 16 h light/8 h dark photoperiod conditions. Adventitious roots from scion were removed regularly. After 7 d of grafting, successfully grafted seedlings were transferred to a MS agar media and grown for another 7 d to explore the cold responsive RNA movement assay.

**Supporting Information Notes S1-S5**

Note S1. Tissue-specific transcriptome profiles of two apple graft combinations.

A total of 48 libraries were obtained from stem vasculature samples of scion and rootstock tissues of ‘Gala’/‘G202’ and ‘Gala’/‘M9’ at three winter stages and used for mapping against 49,944 protein coding genes from apple pan-genome ^18^. A principal component analysis (PCA) was first performed on both rootstock and scion data set consisting of 24 RNA-seq libraries each. In rootstock, PC1 separated the difference of three cold stages, explaining 26.4% of the total variance, followed by the separation of two rootstock genotypes in PC2 accounting for 16.6% of variance **(Fig. 1b)**. On the other hand, in scion PCA, only seasonal difference was separated showing the 34.3% of total variance but the difference between two genotypes in each stage was not explained well in the second component **(Fig. 1c)**.

Note S2. Validation of seasonal flows responsive to cold through SNP analysis.

By mapping of WGS data to the ‘Gala’ haploid genome reference ^18^, we obtained a total of 772,030 SNPs unique to ‘Gala’ scion genotype consisting of 516,674 homozygous and 255,356 heterozygous SNPs. In the rootstock genotypes, there were a total of 3,018,830 SNPs unique to ‘G202’ (1,977,103 homozygous and 1,041,727 heterozygous SNPs) and 2,682,143 SNPs unique to ‘M9’ (457,561 homozygous and 2,224,582 heterozygous SNPs) (**Fig. 4b**). When only SNPs within the 45,352 CDS reference sequence were considered, ‘Gala’ contained only 81,290 SNPs, while there were 250,046 in ‘G202’ and 224,662 in ‘M9’, respectively. This result is expected since ‘Gala’ haploid genome was used for mapping in all cases, and it reflects well the heterozygosity rate of the two rootstock genotypes. Overall, between the two rootstock genotypes, ‘M9’ exhibited more heterozygous than ‘G202’ both genome-wide and within the CDS sequences.

**Note S3. Agroinfiltration assay for mRNA mobility validation in response to cold.**

For target selection, we focused on the upwardly mobile mRNAs at CA stage that contains rootstock genotype-unique SNPs. Given most of vascular flow at late autumn/early winter is formed from shoot to root (source-to-sink) with cold acclimation process, these rootstock-derived mRNAs upwardly mobile toward scion could be closely connected with metabolism that may directly/indirectly mediate scion cold tolerance. Among a total of 31 mobile mRNAs having rootstock-derived SNPs pooled from across all cold stages, 20 were detected at CA stage to contain at least one SNP (**Table S19**). After examining the mobility direction of these mRNAs classified as upwardly mobile in our previous seasonal flow analysis, we found that six mRNAs (*MdTSJT1*, *MdNPF6.4*, *MdHSFA3* and unknown genes Mdg_07g000980, Mdg_09g022000, Mdg_12g014450) were shared with the non-redundant genes of upwardly mobile mRNAs at CA stage, implying the potential rootstock-derived mRNAs upwardly transported to scion against the general source-to-sink flow at early winter during cold acclimation.

**Note S4. Assay for phenotypic assessment of cold tolerance in *Arabidopsis***

To uncover whether *Arabidopsis* *vni2* loss-of-function mutant show cold susceptible phenotype, the cold tolerance was compared among four *Arabidopsis* genotypes: wile-type Col-0 and *vni2*, *CBF3* (cold tolerant) ^19^, *cbfs-1* (cold susceptible) ^20^ mutants (**Fig. S11a**). The *vni2* mutant significantly showed the highest ELR and the lowest survival rate among the four genotypes, implying its cold susceptible phenotype (**Fig. S11b-c**). Interestingly, these results were consistent with the compatible solute measurement. The content of sucrose, glucose, fructose and proline were also measured and compared after 14 d cold treatment among *Arabidopsis* genotypes. These compatible solutes are parameters for cold tolerance as they function as cryoprotectants ^21-23^. Indeed, the *vni2* mutant significantly accumulated proline similar to cold susceptible *cbfs-1*, which level was much less than both wild-type Col-0 and cold tolerant *CBF3* mutant (**Fig. S11d**). Likewise, the accumulation level of three soluble sugars was significantly lower in *vni2* and *cbfs-1* mutants than in the others (**Fig. S11e**). These results indicate the *Arabidopsis* *vni2* mutant shows a cold susceptible phenotype.

**Note S5. Characteristics of metabolic signals involved in seasonal flow between rootstocks and scions.**

After grouping 544 highly-correlated potential mobile signals by movement directions consisting of 199 shared and 438 (199/239) unique (up/down) mobility (**Fig. 3d**), we investigated the expression patterns of enriched genes, regarding the nine functional categories including photosynthesis, secondary metabolism, hormone metabolism, abiotic stress, transcription factors, development, signalling, lipid metabolism, and transport **(Figs. 3e,** **S12**). Of noteworthy, some functional groups were found consistently mobile in all three stages (e.g., hormone metabolism, transcription factors) while others specific to each winter stage and genotype (e.g., secondary metabolism, lipid metabolism).

Apart from its function, photosynthesis is closely related to plant cold response as chloroplasts play a key role responding to external cold stress via ROS signalling ^24^. In our RNA-seq data, photosynthesis-related genes were detected in the groups of downward and contrasting mobility showing the season-specific expression patterns. Even though none of photosynthesis-related genes were detected at any stage in the group unique to upward mobility **(Fig. S12a)**, genes involved in PS subunits (e.g., *PSBQ*, *PSBP*) were rather detected as downwardly mobile commonly in two apple graft combinations during CA stage **(Fig. S12b)**. During DW and DA stages, we found a clear genotypic difference in the direction of contrasting mobility of other genes as upwardly mobile in ‘Gala’/‘G202’ and downwardly mobile in ‘Gala’/‘M9’ **(Fig. S12c)**. At DW, the expression of *PSAE*, *LHCA4* mRNAs were upwardly mobile in ‘Gala’/‘G202’ but downwardly in ‘Gala’/‘M9’, all of which mobility were validated by SNPs as well (**Fig. 4d**). Similarly, *LHCA3* and *FER1* showed the same tendency of contrasting mobility between ‘Gala’/‘G202’ and ‘Gala’/‘M9’ at DA stage. This trend was also found in our GO enrichment results (**Fig. 2b, d**), showing that photosynthesis was enriched in rootstock tissue of cold-tolerant ‘Gala’/‘G202’ compared to ‘Gala’/‘M9’, however the enrichment was rather observed in scion tissue of cold-susceptible ‘Gala’/‘M9’. The rootstock of cold-susceptible ‘Gala’/‘M9’ also exhibited the enrichment of GO term response to stress (GO:0006950) rather than the photosynthesis term (GO:0015979) that was solely enriched in ‘G202’ rootstock. Combined together, these results may imply that the rootstock of cold tolerant ‘Gala’/‘M9’ tended to be more sensitive to external changes in temperature and less de-acclimated to cold, inducing additional stress responsive signals compared to ‘Gala’/‘G202’. Subsequently, instead of rootstock, the scion tissue is rather promoted to generate chloroplast-mediating cold sensing signals in ‘Gala’/‘M9’ whereas the rootstock of ‘Gala’/‘G202’ is still responsible for the same signal.

Next, the secondary metabolism category of highly-correlated DEGs showed several genes related to flavonoid and lignin biosynthesis as seasonally enriched. Flavonoids are known to mediate cold tolerance in Arabidopsis ^25^ and apple ^26,27^. Flavonoid biosynthesis genes were detected to be upwardly regulated in ‘Gala’/‘M9’ compared to ‘Gala’/‘G202’ at DA (e.g., *UGT91C1, CHIL*) **(Fig. S12a)**. As one of main extracellular matrix components in cell wall, lignin is known to mediate the cell wall thickness and integrity in response to cold and osmotic stresses ^28^ and its biosynthesis is positively regulated in vasculature under cold treatment ^29^. We found that among secondary metabolism category, the expression patterns of genes involved in lignin biosynthesis was more upregulated in ‘Gala’/‘G202’ compared to ‘Gala’/‘M9’ showing the upward mobility of *PAL1* at DW and *4CL3* at DA.

In the category of hormone metabolism genes related to auxin, ABA and cytokinin were identified in all three stages separated by each group of mobility direction. Auxin-related genes (e.g., *TSJT1, PIN1, MES17*) was commonly found differentially enriched in both graft combinations across all stages. Previous study reported that auxin signalling is controlled by cold response including the inhibition of auxin transport toward shoot through intracellular trafficking of its efflux carriers in Arabidopsis ^30^. In our data, an auxin responsive *TSJT*1 was upwardly mobile with the relatively high expression in ‘Gala’/‘M9’ than ‘Gala’/‘G202’ during cold acclimation stage. At DW, *PIN1*, an auxin efflux carrier gene, was more downwardly mobile in ‘Gala’/‘G202’ than ‘Gala’/‘M9’. Similarly, the movement of *MES17* mRNA, which mediates auxin homeostasis ^31^, was more downwardly made in ‘Gala’/‘G202’ as well at DA stage. In addition to this, there was auxin-related TF (*IAA24*) also detected at DA among transcription factor group, which showed a tendency of contrasting mobility between ‘Gala’/‘G202’ and ‘Gala’/‘M9’ (**Fig. 12c**). As a member of auxin responsive factors, *IAA24* (also known as *MONOPTEROS*) is known to play a key role in promoting vascular development ^32^. Even though the cold tolerant ‘Gala’/‘G202’ showed downward mobility of *IAA24* mRNA, the movement direction was rather upwardly maintained in cold susceptible ‘Gala’/‘M9’. These results may imply the movement of auxin metabolism is less activated when apple scion is grafted onto cold susceptible rootstock, considering the general shoot-to-root direction of auxin flow to mediate various signals for plant growth ^33^. Furthermore, the downward mobility of auxin metabolism genes in cold susceptible ‘Gala’/‘M9’ could also be complementary to the enrichment of upwardly mobile genes involved in flavonoid biosynthesis regarding the auxin transport can be negatively regulated by flavonoids ^34,35^.

For the genes specific to each cold stage, at CA the expression of *SLD1,* which mediates sphingolipid metabolism, was downwardly mobile in cold susceptible ‘Gala’/‘M9’. In abiotic stress group, genes related to cold response were more enriched in ‘Gala’/‘M9’ than ‘Gala’/‘G202’ in both directions (*GLP5* as upwardly mobile and *Adenine nucleotide alpha hydrolases-like* as downwardly mobile). In transcription factor group, *CAF1K* and *AGL79* were enriched as upwardly mobile specifically to ‘Gala’/‘M9‘ and *RPOC2*, *WLIM2* and *Zinc finger (CCCH-type) family* genes were downwardly mobile in both graft combinations. In development group, the expression pattern of *HSFA3* and *NAC083* showed a contrasting mobility (e.g., *HSAF3* for upward direction in ‘Gala’/‘G202’ and downward in ‘Gala’/‘M9’). *FLZ10*, known to negatively regulate cell growth via SnRK signalling ^36^ was identified as upwardly mobile only detected in ‘Gala’/‘G202’, not in ‘Gala’/‘M9’. In transport group, a nitrate transporter family gene *NPF6.4* was identified as upwardly mobile but relatively more enriched in ‘Gala’/‘M9’. The potassium transporter *KT2* and one of ABC transporter *ABCG28* were downwardly mobile specific to ‘Gala’/‘M9’. Given cold responsive genes (e.g., *GLP5*) were more enriched in ‘Gala’/‘M9’ and the growth inhibiting TF (*FLZ10*) was found in ‘Gala’/‘G202’, these results may imply that during cold acclimation process, apple scion grafted onto cold susceptible rootstock tend to receive more cold responsive signals scion grafted onto cold tolerant rootstock is rather likely to obtain the signal for delaying growth inhibition induced by cold.

At DW, genes mediating heat (*HSP70*) and drought (*QUL2*) stress responses were detected as downwardly mobile in both graft combinations. In transcription factor group, *JKD*, a C2H2 zinc finger family gene, which mediates the meristem development through the regulation of stem cell patterning ^37^, exhibited a contrasting mobility between two graft combinations, (upwardly mobile in ‘Gala’/‘G202’ and downwardly mobile in ‘Gala’/‘M9’). In development group, *HB8*, *BAM3*, *SAG101* were found to have a contrasting mobility between ‘Gala’/‘G202’ and ‘Gala’/‘M9’ and *RmlC-like cupins superfamily* was identified as downwardly mobile in ‘Gala’/‘G202’. In transport group, couple of amino acid transporter genes (*GAT1*, *LYS/HIS transporter 7*) were found as upwardly mobile. Potassium transporters are known to be associated with drought tolerance by regulating ion homeostasis ^38-40^. Interestingly, a couple of potassium transporters were sequentially enriched as downwardly mobile in ‘Gala’/‘M9’ from early to mid-winter including *KT1*.

At DA, the fatty acid synthesis-related gene *ACP* was found as downwardly mobile in ‘Gala’/‘M9’ in the lipid metabolism category. In abiotic stress group, heat stress related genes such as *HSP70* and *SMXL7* were commonly found in both directions but more enriched in ‘Gala’/‘G202’ than ‘Gala’/‘M9’ (*HSP70* as upwardly mobile and *SMXL7* as downwardly mobile). A couple of R2R2-MYB transcription factors *MYB14* and *MYB62* were upwardly mobile in ‘Gala’/‘M9’. In development group, ‘Gala’/‘G202’ showed the enrichment of the upwardly mobile genes (e.g., *Dormancy/auxin associated family*, *NAC029* and *VEP1*) and one downwardly mobile gene (*SWEET2*) in their expressions. In transport group, ‘Gala’/‘G202’ showed the expression of two chloride channel family genes *CLC-B* and *CLC-C* as upwardly mobile and nitrogen transporter *NPF7.3* as downwardly mobile. On the other hand, *SLAC1* and *ABCI20* were found as upwardly mobile in ‘Gala’/‘M9’.

**References**

1 Guak, S. & Neilsen, D. Chill unit models for predicting dormancy completion of floral buds in apple and sweet cherry. *Hortic. Environ. Biotechnol.* **54**, 29-36, doi:10.1007/s13580-013-0140-9 (2013).

2 Anderson, J., Richardson, E. & Kesner, C. in *I International Symposium on Computer Modelling in Fruit Research and Orchard Management 184.* 71-78.

3 Richardson, E. A., Seeley, S. & Walker, D. A model for estimating the completion of rest for" Redhaven" and" Elberta" peach trees. *HortScience* **9**, 331-332 (1974).

4 Fishman, S., Erez, A. & Couvillon, G. The temperature dependence of dormancy breaking in plants: mathematical analysis of a two-step model involving a cooperative transition. *J. Theor. Biol.* **124**, 473-483 (1987).

5 Parkes, H., Darbyshire, R. & White, N. Chilling requirements of apple cultivars grown in mild Australian winter conditions. *Sci. Hortic.* **260**, 108858 (2020).

6 Sun, X. *et al.* Phased diploid genome assemblies and pan-genomes provide insights into the genetic history of apple domestication. *Nat. Genet.* **52**, 1423-1432 (2020).

7 Ferrero, S. *et al.* Transcriptomic signatures in seeds of apple (Malus domestica L. Borkh) during fruitlet abscission. *PLoS One* **10**, e0120503 (2015).

8 Howe, E. A., Sinha, R., Schlauch, D. & Quackenbush, J. RNA-Seq analysis in MeV. *Bioinformatics* **27**, 3209-3210 (2011).

9 Tian, T. *et al.* agriGO v2. 0: a GO analysis toolkit for the agricultural community, 2017 update. *Nucleic Acids Res.* **45**, W122-W129 (2017).

10 Daccord, N. *et al.* High-quality de novo assembly of the apple genome and methylome dynamics of early fruit development. *Nat. Genet.* **49**, 1099-1106 (2017).

11 Sherman, B. T. *et al.* DAVID: a web server for functional enrichment analysis and functional annotation of gene lists (2021 update). *Nucleic Acids Res.* **50**, W216-W221 (2022).

12 Kanehisa, M. & Goto, S. KEGG: kyoto encyclopedia of genes and genomes. *Nucleic Acids Res.* **28**, 27-30 (2000).

13 Bowen, J. *et al.* Selection of low-variance expressed Malus x domestica (apple) genes for use as quantitative PCR reference genes (housekeepers). *Tree Genet. Genom.* **10**, 751-759 (2014).

14 Tellmann, G. & Geulen, O. LightCycler® 480 Real-Time PCR system: innovative solutions for relative quantification. *BIOCHEMICA-MANNHEIM-* **4**, 16 (2006).

15 Ranallo-Benavidez, T. R., Jaron, K. S. & Schatz, M. C. GenomeScope 2.0 and Smudgeplot for reference-free profiling of polyploid genomes. *Nat. Commun.* **11**, 1432 (2020).

16 Lohse, M. *et al.* M ercator: a fast and simple web server for genome scale functional annotation of plant sequence data. Report No. 0140-7791, (Wiley Online Library, 2014).

17 Bates, L., Waldren, R. a. & Teare, I. Rapid determination of free proline for water-stress studies. *Plant Soil* **39**, 205-207 (1973).

18 Sun, X. *et al.* Phased diploid genome assemblies and pan-genomes provide insights into the genetic history of apple domestication. *Nat. Genet.* **52**, 1423-1432, doi:10.1038/s41588-020-00723-9 (2020).

19 Liu, Z. *et al.* Plasma membrane CRPK1-mediated phosphorylation of 14-3-3 proteins induces their nuclear import to fine-tune CBF signaling during cold response. *Mol. Cell* **66**, 117-128. e115 (2017).

20 Jia, Y. *et al.* The cbfs triple mutants reveal the essential functions of CBF s in cold acclimation and allow the definition of CBF regulons in Arabidopsis. *New Phytol.* **212**, 345-353 (2016).

21 Zuther, E., Juszczak, I., Ping Lee, Y., Baier, M. & Hincha, D. K. Time-dependent deacclimation after cold acclimation in Arabidopsis thaliana accessions. *Sci. Rep.* **5**, 12199 (2015).

22 Rohde, P., Hincha, D. K. & Heyer, A. G. Heterosis in the freezing tolerance of crosses between two Arabidopsis thaliana accessions (Columbia‐0 and C24) that show differences in non‐acclimated and acclimated freezing tolerance. *Plant J.* **38**, 790-799 (2004).

23 Klotke, J., Kopka, J., Gatzke, N. & Heyer, A. Impact of soluble sugar concentrations on the acquisition of freezing tolerance in accessions of Arabidopsis thaliana with contrasting cold adaptation–evidence for a role of raffinose in cold acclimation. *Plant, Cell Environ.* **27**, 1395-1404 (2004).

24 Gan, P., Liu, F., Li, R., Wang, S. & Luo, J. Chloroplasts—beyond energy capture and carbon fixation: tuning of photosynthesis in response to chilling stress. *Int. J. Mol. Sci.* **20**, 5046 (2019).

25 Schulz, E., Tohge, T., Zuther, E., Fernie, A. R. & Hincha, D. K. Flavonoids are determinants of freezing tolerance and cold acclimation in Arabidopsis thaliana. *Sci Rep* **6**, 34027, doi:10.1038/srep34027 (2016).

26 Song, T. *et al.* Identification of new regulators through transcriptome analysis that regulate anthocyanin biosynthesis in apple leaves at low temperatures. *PloS one* **14**, e0210672 (2019).

27 An, J. P. *et al.* An apple MYB transcription factor regulates cold tolerance and anthocyanin accumulation and undergoes MIEL1-mediated degradation. *Plant Biotechnol. J.* **18**, 337-353, doi:10.1111/pbi.13201 (2020).

28 Ji, H. *et al.* The Arabidopsis RCC1 Family Protein TCF1 Regulates Freezing Tolerance and Cold Acclimation through Modulating Lignin Biosynthesis. *PLoS Genet.* **11**, e1005471, doi:10.1371/journal.pgen.1005471 (2015).

29 Shafi, A., Dogra, V., Gill, T., Ahuja, P. S. & Sreenivasulu, Y. Simultaneous over-expression of PaSOD and RaAPX in transgenic Arabidopsis thaliana confers cold stress tolerance through increase in vascular lignifications. *PloS one* **9**, e110302 (2014).

30 Shibasaki, K., Uemura, M., Tsurumi, S. & Rahman, A. Auxin response in Arabidopsis under cold stress: underlying molecular mechanisms. *The Plant Cell* **21**, 3823-3838 (2009).

31 Yang, Y. *et al.* Inactive methyl indole-3-acetic acid ester can be hydrolyzed and activated by several esterases belonging to the At MES esterase family of Arabidopsis. *Plant Physiol.* **147**, 1034-1045 (2008).

32 Hardtke, C. S. & Berleth, T. The Arabidopsis gene MONOPTEROS encodes a transcription factor mediating embryo axis formation and vascular development. *The EMBO journal* **17**, 1405-1411 (1998).

33 Petrášek, J. & Friml, J. Auxin transport routes in plant development. *Development* **136**, 2675-2688 (2009).

34 Kuhn, B. M., Geisler, M., Bigler, L. & Ringli, C. Flavonols accumulate asymmetrically and affect auxin transport in Arabidopsis. *Plant Physiol.* **156**, 585-595 (2011).

35 Besseau, S. *et al.* Flavonoid accumulation in Arabidopsis repressed in lignin synthesis affects auxin transport and plant growth. *The Plant Cell* **19**, 148-162 (2007).

36 Jamsheer K, M. *et al.* FCS‐like zinc finger 6 and 10 repress Sn RK 1 signalling in Arabidopsis. *Plant J.* **94**, 232-245 (2018).

37 Welch, D. *et al.* Arabidopsis JACKDAW and MAGPIE zinc finger proteins delimit asymmetric cell division and stabilize tissue boundaries by restricting SHORT-ROOT action. *Genes Dev.* **21**, 2196-2204 (2007).

38 Li, W., Xu, G., Alli, A. & Yu, L. in *Semin. Cell Dev. Biol.* 133-141 (Elsevier).

39 Rajappa, S., Krishnamurthy, P. & Kumar, P. P. Regulation of AtKUP2 expression by bHLH and WRKY transcription factors helps to confer increased salt tolerance to Arabidopsis thaliana plants. *Front. Plant Sci.* **11**, 1311 (2020).

40 Nieves-Cordones, M., Caballero, F., Martínez, V. & Rubio, F. Disruption of the Arabidopsis thaliana inward-rectifier K+ channel AKT1 improves plant responses to water stress. *Plant Cell Physiol.* **53**, 423-432 (2012).
